# Dual inhibition of mTOR and HSP90 enhances cisplatin efficacy and overcomes resistance in ovarian cancer

**DOI:** 10.1038/s41419-026-08533-3

**Published:** 2026-03-27

**Authors:** Rita Lombardi, Laura Addi, Biagio Pucci, Francesca Bruzzese, Maura Sonego, Anna Nespolo, Maria Serena Roca, Federica Iannelli, Luigi Alfano, Francesca Capone, Elena Di Gennaro, Gustavo Baldassarre, Alfredo Budillon

**Affiliations:** 1 Experimental Animal Unit, Istituto Nazionale Tumori Fondazione G. Pascale - IRCCS, Naples, Italy; 2 Experimental Pharmacology Unit, Istituto Nazionale Tumori Fondazione G. Pascale - IRCCS, Naples, Italy; 3 Molecular Oncology Unit, Centro di Riferimento Oncologico di Aviano (CRO) IRCCS, National Cancer Institute, Aviano (PN), Italy; 4 Breast Unit, Istituto Nazionale Tumori Fondazione G. Pascale - IRCCS, Naples, Italy; 5 Scientific Directorate, Istituto Nazionale Tumori Fondazione G. Pascale - IRCCS, Naples, Italy; 6 Present Address: Laboratorio di Patologia Clinica, Azienda Ospedaliera di Rilievo Nazionale e di Alta Specialità San Giuseppe Moscati, Avellino, Italy

**Keywords:** Ovarian cancer, Phosphorylation

## Abstract

Epithelial ovarian cancer (EOC) represents the most lethal gynecological disease, with a 5-year relative survival rate of 46% after the diagnosis. Standard treatment includes surgery followed by platinum (Pt)-based chemotherapy. However, Pt-resistance frequently occurs and strongly impact on the survival of EOC patients for whom we still do not have valid therapeutic options. By using a proteomic approach, we previously demonstrated a potential role of HSP90 in the mechanism of resistance in vitro, ex vivo e partially in vivo. To further investigate in depth the mechanism by which EOC cells acquired Pt-resistance, we used a quantitative phosphoproteomics approach followed by enrichment functional analysis. Here, we identified 542 differentially expressed phosphoproteins in Pt-resistant compared to parental cells identifying mTOR and HSF1 as the most enriched pathways. The up-regulation of the phosphorylated form of PDK1, AKT, mTOR, and RPS6 was observed in Pt-resistant compared to parental cells. Moreover, we also demonstrated the up-regulation of the activity of HSF1 along with the elevation crucial components of the chaperone complex machinery HSP90, HSP70 and HSP40. Since mTOR is an attractive target for therapeutic intervention because of its key role in the crosstalk of various signaling pathways, we propose a novel therapeutic strategy based on the pharmacologic inhibition of HSP90 and mTOR able to further potentiate the Pt-based chemotherapy. Accordingly, the combination of ganetespib (an HSP90 inhibitor) and temsirolimus (a FDA approved-mTOR inhibitor) with cisplatin synergistically reduced colony formation and microtissues cell growth in vitro by increasing DNA-damage and apoptosis and in vivo enhancing mouse survival. Mechanistically, the triple combination treatment, impaired the proteins involved in mTOR signaling and HSF1 transactivation. Notably, all these data were confirmed also in Pt-resistant Non Small Cell Lung Cancer models. Collectively, our findings identify a promising new antitumor strategy for the treatment of Pt-resistance in cancer patients.

## Introduction

The majority of cancer patients are still treated with chemotherapy (CT), either alone or in combination with radiotherapy, targeted therapies or immunotherapy [1]. Platinum (Pt)-based drugs, including cisplatin (CDDP), carboplatin and oxaliplatin, are widely used in anticancer therapies [2]. To date, it is estimated that approximately 50% of all patients with cancer are treated with CDDP with significant clinical successes either when it is used as monotherapy, or in combination treatments [3]. In details, among others, CDDP-based therapies are approved for the treatment of epithelial ovarian cancer (EOC), non-small cell lung cancer (NSCLC) and head and neck squamous cell carcinoma (HNSCC) patients [4, 5]. Unfortunately, relapses of acquired drug-resistant disease are common in advanced stages and have hampered CDDP clinical utility. Therefore, improving the clinical efficacy of CDDP and other Pt-based drugs has emerged as a critical unmet need for anticancer approaches and as a central goal for researchers worldwide in the fields of oncology and pharmacology.

EOC, which can be divided into high grade-serous ovarian cancer, endometrioid carcinomas, clear-cell carcinomas, mucinous carcinomas, and low-grade serous carcinomas represents the third most common and the most lethal gynecologic malignant neoplasm worldwide with a 5-year relative survival rate of 46% after the diagnosis [6]. EOC are clinically classified as Pt-sensitive (∼80% of the cases) and Pt-resistant disease based on the timing of response to first-line Pt-based therapy. For Pt-sensitive patients the addition of targeted therapies as concomitant or maintenance treatments has become a standard component of first-line therapies and significantly improved patients’ survival [7, 8]. However, more than half of all originally Pt-sensitive EOC patients experience Pt-resistant recurrences, that predicts poor overall survival and for which no effective treatments are available [9]. Indeed, also immunotherapies, that is efficacious in treatment of many other solid malignant neoplasms, have shown less promise for Pt-resistant EOC patients to date [10].

Pt-based chemotherapy is widely used as standard of care in different treatment setting for NSCLC patients lacking drug-targetable driver mutations (approximately 85–90%) [11]. Similarly, CDDP is probably the most widely used agent to treat HNSCC [12].

As observed in EOC also for NSCLC and HNSCC patients, the development of Pt-resistance predict slow survival rates and represents a pressing unmet clinical need.

Consequently, many studies focus on targeted approaches to understand the molecular mechanisms associated with the onset of Pt-resistance, with more attempts to the ovarian cancer treatment landscape [13].

The principal cytotoxic mechanism of CDDP involves the binding of Pt to DNA after its entry into tumor cells. Within the tumor cell, several mechanisms contribute to Pt-resistance, including reduced drug uptake, enhanced drug efflux, increased DNA damage repair, and inactivated cell death signaling [14]. Our group has been studying for many years the molecular mechanisms underlying Pt-response and resistance and recently, using a proteomic approach, we have characterized the proteome of three Pt-resistant (Pt-res) isogenic high-grade EOC cell lines (TOV-112D Pt-res, MDAH-2774 Pt-res, and OVSAHO Pt-res) in comparison to their parental Pt-sensitive counterparts [15]. In detail, we identified the heat-shock protein 90 (HSP90) as a key player in the network of proteins differentially expressed between resistant and parental cells and demonstrated that the HSP90 inhibitor ganetespib exhibited a potent antitumor synergistic effect with CDDP in Pt-res EOC cells both in vitro, ex vivo, and partially in vivo [15]. Notably, we obtained similar results in a Pt-res NSCLC isogenic model [16], suggesting that the same molecular mechanisms can operate in different tumor types treated with Pt-based therapies.

Here, to further investigate in depth the mechanisms underlying the acquired Pt-resistance in EOC, we took advantage of a phosphoproteomics approach highlighting Heat shock factor (HSF1) and mammalian target of rapamycin (mTOR) as main activated pathways in our EOC Pt-res cellular models. Our data suggest the efficacy of a novel therapeutic strategy based on the combined pharmacologic inhibition of HSP90 and mTOR to further potentiate Pt-based chemotherapy and to revert Pt-resistance in EOC, NSCLC and HNSCC cells, substantiating the potential existence of a common mechanism across various tumor types.

## Results

### Phosphoproteomic analysis identified several differentially expressed proteins between Platinum-resistant and parental EOC cells

Phosphorylation is one of the most relevant protein post-translational modifications and plays critical roles in the regulation of many cellular processes, including cell cycle, growth, apoptosis, and signal transduction pathways [17].

Based on the notion that Pt-resistant (Pt-res) cells differ from the Pt-sensitive counterpart in their total proteome, we decided to investigate more in depth these differences exploiting a LC-MS/MS phosphoproteomics approach to compare the expression profile of TOV-112D platinum resistant cells (TOV-112D Pt-res cl.7) to the sensible counterpart (TOV-112D) [18–20].

**Fig. 1:**
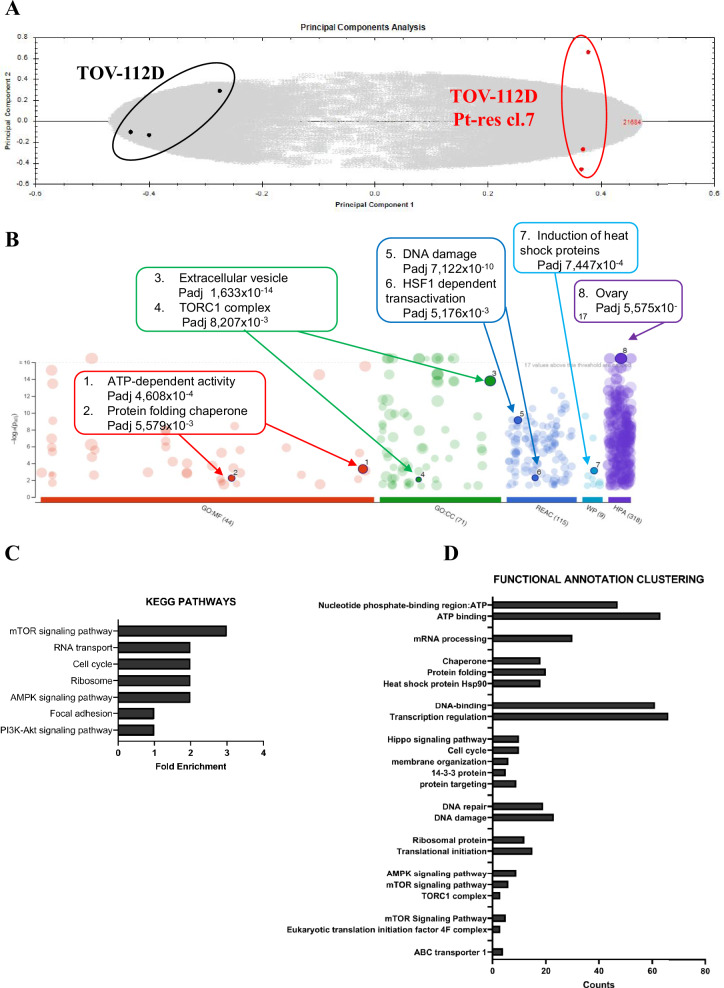
Label-free LC-MS/MS-based phosphoproteomics quantification and bioinformatics analysis. **A** Unsupervised multivariate analysis (PCA plot) showed good experimental reproducibility as demonstrated by the close relation between the three biological replicates of parental (TOV-112D) and resistant cells (TOV-112D Pt-res cl.7). The six spot maps for each cell line clearly clustered into two groups corresponding to Pt-res cells (red) *vs* parental cells (black; *P* < 0.05). *P*-values were calculated based on unpaired Student’s *t* test. **B** Gene set enrichment analysis using g:Profiler (https://biit.cs.ut.ee/gprofiler/). The significantly changed terms enriched by Gene Ontology (GO), Reactome (REAC), Wikipathways (WP) and Human Protein Atlas (HPA). **C**, **D** Functional enrichment analysis of the significant proteins using DAVID software in terms of KEGG pathways and functional annotation clustering.

Principal component analysis (PCA) showed a good experimental reproducibility of phosphoproteomics results as demonstrated by the close relationship between the biological replicates, for each cellular model, into two groups corresponding to TOV-112D Pt-res cl.7 cells (red) versus (*vs*) parental cells (black) (Fig. 1A). Accordingly, the technical variability of each peptide/protein estimated using the Pearson correlation coefficients (Perseus software, v. 1.6.6.0), demonstrated high peptides/proteins reproducibility among biological replicates in parental (TOV-112D #1 vs TOV-112D #2) and resistant cells (TOV-112D Pt-res cl.7 #1 vs TOV-112D Pt-res cl.7 #2) while, TOV-112D #1 vs TOV-112D Pt-res cl.7 #1 had a lower correlation coefficient. (Supplementary Fig. 1A).

The analyses of 542 phosphoproteins using the Progenesis software (see methods for details) allowed the identification of proteins differentially expressed between sensitive and resistant cells (data availability statement 10.5281/zenodo.16564396) and their classification according to the gene ontology (GO) database and pathway analysis. Both g-Profiler and DAVID software used for data analyses highlighted mTOR as one of the main differentially expressed pathways (Fig. 1B–D and Supplementary Fig. 1B).

In accord with a possible pivotal involvement of mTOR pathway in differentiate Pt-sensitive and resistant cells we also observed HSF1 dependent transactivation and protein folding chaperone and PI3K/AKT signaling pathways as differentially enriched in the two models (Fig. 1B, C) [21].

Finally, functional annotation clustering showing the number of proteins related to the ontology categories (DAVID software) and volcano plot analyses confirmed the increase of mTOR, HSF1 and HSP90 activity in TOV-112D resistant cells **(**Fig. 1D and Supplementary Figure 1C**)**.

**Fig. 2:**
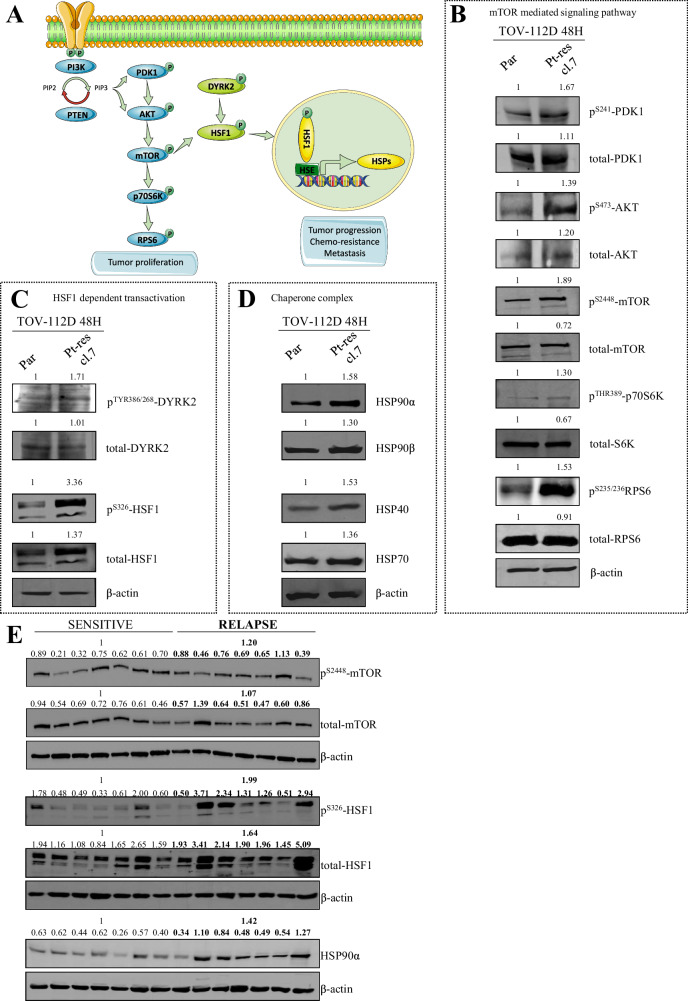
Schematic representation of the main investigated pathways and western blot validation experiments of the identified phosphoproteins. **A** Upon receptor binding by growth factors and cytokines, phosphatidylinositol 3-kinase (PI3K) is activated and in turn phosphorylates PDK1 and AKT, witch activate mTOR by phosphorylation. mTOR phosphorylates downstream p70S6K which activates RPS6 promoting tumor proliferation. Moreover, mTOR or DYRK2 directly activates HSF1 protein by phosphorylation, promoting its nuclear translocation and transcription of HSP genes. **B** Western blot analysis, performed after 48 h of cell culture, showed an activation of mTOR mediated signaling pathway thorough the phosphorilation of p^S241^-PDK1, p^S473^-AKT, p^S2448^-mTOR, p^THR389^-p70S6K, p^S235/236^RPS6 proteins in TOV-112D Pt-res cl.7 resistant cells *vs* TOV-112D parental cells (Par). Densitometric analysis was done by ImageJ software and reported as the ratio: phospho-protein/total protein/loading control (ß-actin). **C** HSF1 dependent transactivation was confirmed by western blot analysis performed after 48 h of cell culture, showing an upregulation of p^TYR386/268^-DYRK2 and p^S326^-HSF1 proteins in TOV-112D Pt-res cl.7 resistant cells compared with TOV-112D parental cells (Par). Densitometric analysis was done by ImageJ software and reported as ratio: phospho-protein/total protein/loading control (ß-actin). **D** Expression of the proteins of chaperone complex (HSP90α, HSP90β, HSP40, HSP70) was evaluated by western blot experiment in TOV-112D Pt-res cl.7 resistant cells *vs* TOV-112D parental cells (Par) performed after 48 h of cell culture. Densitometric analysis was done by ImageJ software and reported as ratio relative to the indicated loading control (ß-actin). **E** Expression of p^S2448^-mTOR, p^S326^-HSF1 and HSP90α was evaluated by western blot experiment in Pt-resistant primary cells compared to Pt-sensitive primary cells performed after 48 h of cell culture. Densitometric analysis values are reported as ratios relative to the corresponding β-actin levels.

The PI3K/AKT/mTOR pathway **(**Fig. 2A**)** is a central hub involved in the regulation of proliferation, progression, metastasis and chemo-resistance of tumor cells [22], and several lines of evidence suggest that the mTOR pathway is intricately linked to the activity of the transcription factor HSF1. Specifically, mTOR could directly phosphorylate HSF1, promoting its activation, nuclear translocation and heat shock proteins transcription [21].

Therefore, based on our phospho-proteomic data and the current literature we decided to better investigate the role of PI3K/mTOR/HSF1 axis in Pt-sensitive and resistant TOV-112D cells trying to understand if this axis could represent a specific vulnerability of Pt-res tumors.

First, we validated phospho-proteomic data using western blot analyses confirming that TOV-112D Pt-res cl. 7, compared to parental cells, had increased expression of the phosphorylated forms of PDK1, AKT, mTOR, p70S6K and RPS6, all protein members of the PI3K/mTOR signaling pathway (Fig. 2B). Moreover, TOV-112D Pt-res cells showed an increased expression of the activating phosphorylation of HSF1 and of its targets heat shock proteins HSP90, HSP70 and HSP40, which are crucial components of the protein folding chaperone complex (Fig. 2C, D). Interestingly, among the differentially expressed proteins, we identified by phosphoproteomics approach and validated by western blot the kinase DYRK2 known as client of HSP90 and able to phosphorylate HSF1 (Fig. 2C) [23].

Importantly, the expression of HSP90, phosphorylated HSF1, and with a lesser extent phosphorylated mTOR were confirmed in primary culture obtained from Pt-sensitive and Pt-res EOC patients, confirming the clinical relevance of the preclinical results (Fig. 2E).

These data are in complete accord with our previous observation indicating that HSP90 is a critical hub within a network of differentially expressed proteins identified in a Pt-res non-small cell lung cancer (NSCLC) isogenic model (A549 CPr) [16].

Indeed, NSCLC Pt-res model cells A549 CPr showed an hyper-activation of the protein involved in mTOR mediated signaling pathway, HSF1 dependent transactivation, and chaperone complex when compared with the Pt-sensitive A549 counterpart (Supplementary Fig. 2A–C), hypothesizing that similar mechanisms are present in different tumor types.

Based on our previous results demonstrating that HSP90 inhibition synergistically improve the antitumor activity of CDDP in vitro and ex vivo in primary cultures derived from the ascites of Pt-res EOC patients [15], we exploited the CRISPR-Cas9 system to generated knockout HSP90α cells (A549 CPr KO#1, #3, #4) from A549 CPr (Supplementary Fig. 3A). Using these tools we confirmed in a NSCLC model that HSP90α knockout partially reversed Pt-resistance, as verified by colorimetric cytotoxicity assay (Supplementary Fig. 3), colony formation assay (Supplementary Fig. 3C), and induction of apoptosis evaluated by PARP cleavage (Supplementary Fig. 3D).

**Fig. 3:**
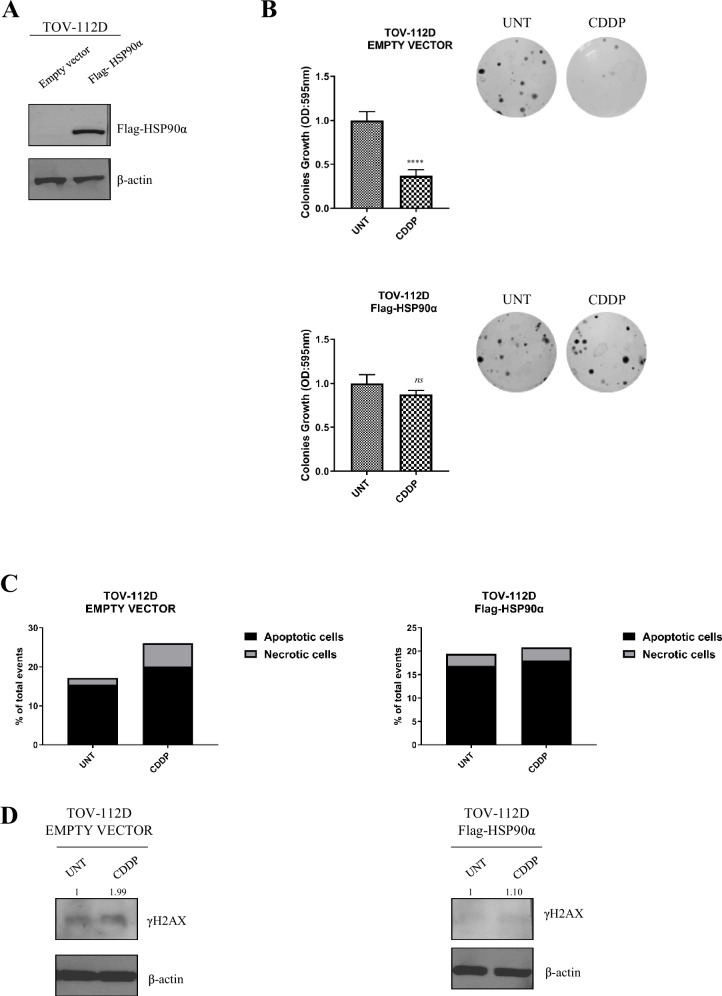
Effect of HSP90α knock-in in Platinum-sensitive TOV-112D cells. **A** Western blot analysis evaluating the expression of Flag-HSP90α in TOV-112D empty vector and TOV-112D over expressing HSP90α (Flag-HSP90α). β-Actin was used as loading control. Western blot quantification was performed by ImageJsoftware. **B** Clonogenic assay of TOV112-D empty vector and TOV-112D Flag-HSP90α cells treated with CDDP at the IC_10_^96h^ for parental cells. Representative data of at least three independent experiments performed in triplicates. Statistically significant results are reported (****P < 0.0001, ns, not statistically significant). **C** Apoptosis and necrosis evaluated by flow cytometry after Annexin V-FITC and propidium iodide staining in TOV-112D empty vector and and TOV-112D Flag- HSP90α, untreated or treated with CDDP for 48 h with CDDP at IC_50_^96h^ doses of parental cells. **D** Western blot analysis of γH2AX expression in TOV-112D empty vector and TOV-112D Flag-HSP90α, untreated or treated with CDDP for 48 h at IC_50_^96h^ doses of parental cells.

Complementary to the knockout studies, we performed HSP90α knock-in experiments (Supplementary materials and methods) in the Pt-sensitive TOV-112D cell lines (Fig. 3A). Clonogenic assays revealed that HSP90α-overexpressing cells exhibited reduced sensitivity to CDDP compared to empty vector controls (Fig. 3B). Moreover, these cells did not display induction of apoptosis or DNA damage upon CDDP treatment, as evaluated by Annexin V staining (Fig. 3C) and γH2AX Western blot analyses (Fig. 3D). These results further support the pivotal role of HSP90 in modulating Pt-resistance. Overall, the collected data support the possibility that acquired Pt-resistance induce a tumor agnostic upregulation of the PI3K/mTOR/HSF1 axis that leads to the overexpression of HSPs chaperone proteins and that this axis might represent a specific vulnerability of Pt-res cells.

### HSP90 and mTOR inhibitors potentiate CDDP antitumor effect and reverse Platinum-resistance

Our present and previous data support the notion that inhibition of HSP90 protein ameliorate the activity of platinum in EOC in vitro also in the context of Pt-res disease but also demonstrated that the platinum plus ganetespib is only partially active in vivo in Pt-res EOC xenograft models [15]. This evidence is in line with the results of clinical trial using ganetespib as a single agent or in combination with chemotherapy in different type of human cancers, showing no improvement of patient survival [24–26]. In the context of Pt-res EOC patients the use of ganetespib in combination with chemotherapy is feasible, although with limited clinical utility even if we have to wait for the results of the activity GANNET53 Phase I/II trial, which will give a final answer on its clinical activity [27, 28].

These preclinical and clinical data suggest that, although HSP90 inhibitors are manageable drugs with potential clinical activity, novel therapeutic strategies should be tested to improve its efficacy toward its clinical translation in the context of chemo-resistant tumors.

Our data showing high levels of mTOR in Pt-res relative to parental cells and the wide use of mTOR inhibitors in several type of cancer with discrete success and manageable toxicities, suggested us that it might also represent a potential therapeutic target in Pt-res EOC setting.

Based on these considerations, we explored the efficacy of a novel therapeutic strategy through the pharmacologic inhibition of HSP90 and mTOR both putatively able to improve the Pt-based chemotherapy.

To this aim, we evaluated the combination of the inhibitors of HSP90 (ganetespib) and mTOR (temsirolimus) plus CDDP in Pt-sensitive and resistant EOC and NSCLC models. We first tested the sensitivity of cell lines to single agent treatment, demonstrating a cross-resistance of Pt-res cells to ganetespib and temsirolimus (Supplementary Table 1).

**Fig. 4:**
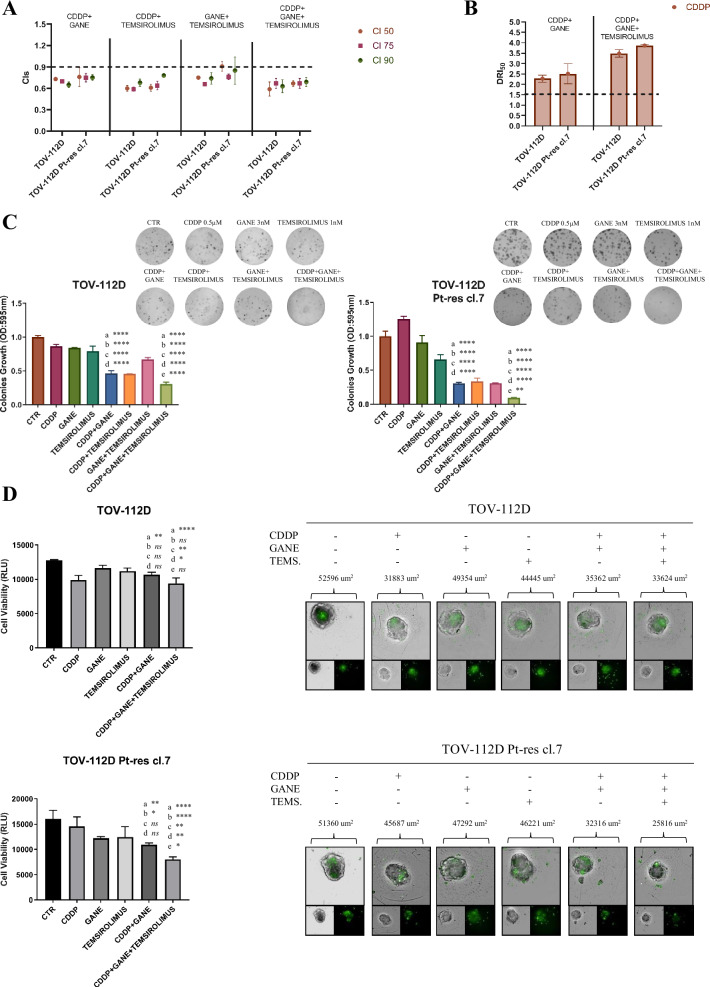
Potentiation of CDDP antitumor effect induced by ganetespib and temsirolimus in parental and Platinum-resistant TOV-112D cells. **A** CI (combination index) values (mean ± SD from at least three separate experiments performed in quadruplicates) computed at 50% (CI50), 75% (CI75) and 90% (CI90) of cell kill by CalcuSyn software after 96 h for TOV-112D and TOV-112D Pt-res cl.7 cells. The treatments are indicated in the figure. The combinations were considered synergistic when CIs were below 0.9, additive when CIs were between 0.9 and 1.1 or antagonism when Cis were more than 1.1. **B** DRI (doses reduction index) values (mean ± SD) for CDDP from at least three separate experiments performed in quadruplicate) that represent the order of magnitude (fold) of dose reduction obtained for IC50 (DRI50) in combination setting compared with each drug alone in TOV-112D and TOV-112D Pt-res cl.7 cells. **C** Synergistic inhibition of colony formationin TOV-112D and TOV-112D Pt-res cl.7 treated with CDDP, ganetespib, temsirolimus or their combination (simultaneous exposure) at the IC_10_^96h^ doses for parental cells. Representative data of at least three independent experiments performed in triplicates. Statistically significant results calculated with one-way ANOVA test are reported (a indicates control group, b indicates CDDP-treated cells, and c indicates ganetespib-treated cells, d indicates temsirolimus-treated cells, e indicates cddp plus ganetespib-treated cells *P < 0.05, **P < 0.01, ***P < 0.001 and ****P < 0.0001, ns, not statistically significant). **D** Synergistic inhibition of microtissues formation by CDDP, ganetespib, temsirolimus alone and in combination. Cancer cells (red ones-marked by cell tracker) and mice fibroblast cells NIH/3T3 were plated in each well and after 24 h treated withIC_50_^96h^ doses of parental cells. Representative images from Opera Phenix confocal microscopy. The graphics represent the number of viable cells in 3D cell culture based on quantitation of the ATP content. Results were obtained by a single experiment performed in triplicate ( ± SD). Statistically significant results calculated with one-way ANOVA test are reported (a indicates control group, b indicates CDDP-treated cells, and c indicates ganetespib-treated cells, d indicates temsirolimus-treated cells, e indicates CDDP plus ganetespib-treated cells *P < 0.05, **P < 0.01, ***P < 0.001 and ****P < 0.0001, ns not statistically significant).

We then investigated the combination of CDDP with ganetespib and/or temsirolimus using equipotent doses and evaluated the CI values calculated at 50% (CI50), 75% (CI75) and 90% (CI90) of cell lethality, in TOV-112D parental and resistant cells. By considering strong synergism CI ≤ 0.8, synergism CI ≤ 0.9, additivity CI > 0.9 and ≤1.1, and antagonism CI > 1.1 [29], we obtained consistent synergistic antiproliferative effects and strongest effects using the triple combination (Fig. 4A and Supplementary Table 2).

The synergistic interaction between the three drugs was then confirmed by evaluating the doses reduction index (DRIs) for CDDP, which represent the order of magnitude (fold) of dose reduction obtained for the IC50 (DRI_50_) of each agent in combination *vs* single drug treatments (Fig. 4B). Importantly, we extended our analysis to an additional EOC model, OVCAR8 and its platinum-resistant counterpart (OVCAR8 Pt.res cl.2), which represents the high-grade serous ovarian cancer subtype, the most prevalent histological form of the disease. We obtained consistent synergistic antiproliferative effects and strongest effects using the triple combination (Supplementary Fig. 4 and Supplementary Table 2). These results further confirmed that concurrent inhibition of HSP90 and mTOR enhances the efficacy of Pt-based chemotherapy. Moreover, we demonstrated that concomitant treatment with CDDP/ganetespib/temsirolimus completely suppressed colony formation in TOV-112D parental and Pt-res cells using the IC_10_^96h^ dosages of parental cells (Fig. 4C, Supplementary Fig. 5A and Supplementary materials and methods).

Although two-dimensional (2D) monolayer in vitro models are time and cost efficient, they lack the complexity of intact physiological systems and may not provide results that can be correlated with in vivo responses [30].

Three-dimensional (3D) in vitro platforms provide a unique alternative to bridge the gap between traditional 2D in vitro and in vivo models [31].

On this regard, we showed by 3D microtissue assay, using NIH/3T3 fibroblast cell line isolated from mice as scaffold and ovarian cancer cells as tumor component, that CDDP/ganetespib/temsirolimus combination led to a potent microtissues regression especially in TOV-112D Pt-res cl. 7 cells, compared to single and double treatments using IC_50_^96h^ dosages of parental cells (Fig. 4D).

**Fig. 5:**
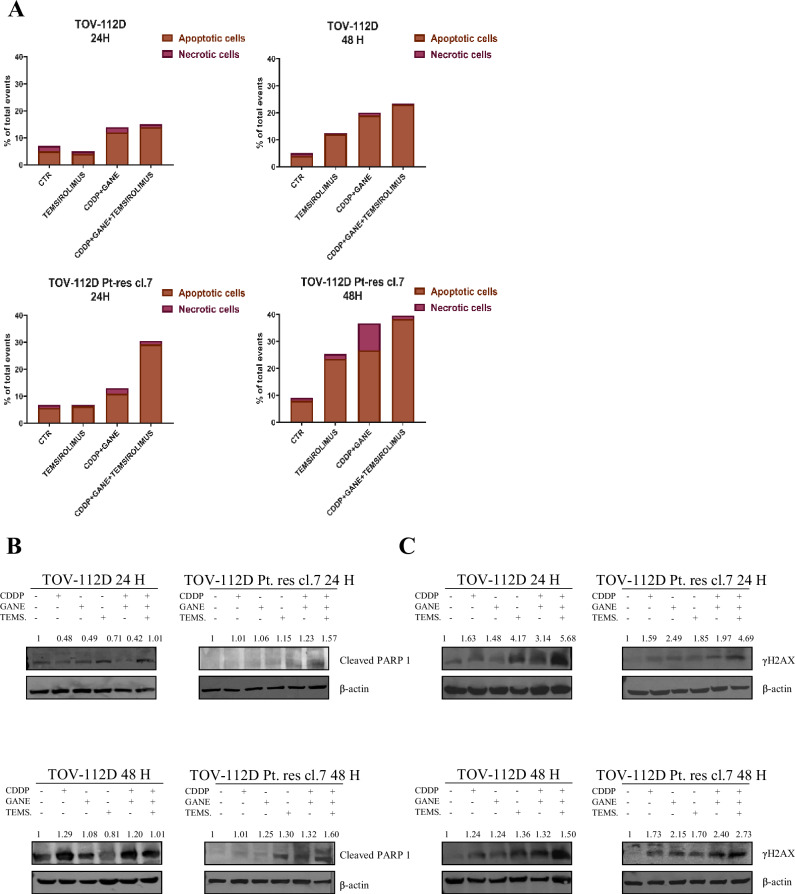
Pro-apoptotic and DNA damage effect of ganetespib and temsirolimus plus CDDP in EOC cells. **A** Apoptosis and necrosis evaluated by flow cytometry after Annexin V-FITC and propidium iodide staining in TOV-112D and TOV-112D Pt-res cl. 7, untreated or treated for 24 h or 48 h, with temsirolimus, CDDP plus ganetespib and CDDP plus ganetespib and temsirolimus at IC_50_^96h^ doses of parental cells. **B** Western blot analysis of cleaved PARP1 in TOV-112D and TOV-112D Pt-res cl. 7 cells untreated or treated with CDDP, ganetespib, temsirolimus and their combinationat IC_50_^96h^ doses of parental cells at the time indicated above. β-actin expression serves as loading control. Western blot quantification was performed by ImageJ software. **C** Western blot analysis of γH2AX in TOV-112D and TOV-112D Pt-res cl. 7 cells untreated or treated with CDDP, ganetespib, temsirolimus and their combination at IC_50_^96h^ doses of parental cells at the time indicated above. β-actin expression serves as loading control. Western blot quantification was performed by ImageJ software.

We observed that the potentiation of CDDP antitumor effect by ganetespib plus temsirolimus was likely due to an increased apoptosis as demonstrated by the evaluation of Annexin V positivity cells (Fig. 5A, Supplementary Fig. 5B and Supplementary materials and methods) and of cleaved PARP1 (Fig. 5B and Supplementary Fig. 5C) expression. The increase apoptosis was accompanied by a clear induction of DNA damage when the CDDP/ganetespib/temsirolimus was used (i.e., γH2AX expression) compared to single and double treatments using IC_50_^96h^ dosages of parental cells (Fig. 5C and Supplementary Fig. 5D).

Notably, similar synergism by the combination of ganetespib and temsirolimus plus CDDP was observed in A549 CPr model, particularly when the triple combination was tested (Supplementary Fig. 6A and Supplementary Table 3). The DRI_50_ evaluation for CDDP and the colony formation assays using IC_10_^96h^ dosages of parental cells confirmed these (Supplementary Fig. 6B, C). Also in the NSCLC model, the anticancer activity of the triple combination was associate to an increase in apoptosis as showed by the evaluation of Annexin V positivity (Supplementary Fig. 6D).

Finally, we demonstrated the synergistic anti-proliferative effect of the triple combination in Head and Neck intrinsic Pt-res Cal27 and Cal33 cell lines, evaluating first the sensitivity of cell lines to single agent treatment (Supplementary Tables 4, 5 and Supplementary Fig. 7A, B). Notably, 3D visualization of the Cal27-GFP + /Luc+ cells highlighted the impact of all treatments on cell mean volume, particularly of the triple combination (Supplementary Fig. 7C).

**Fig. 6:**
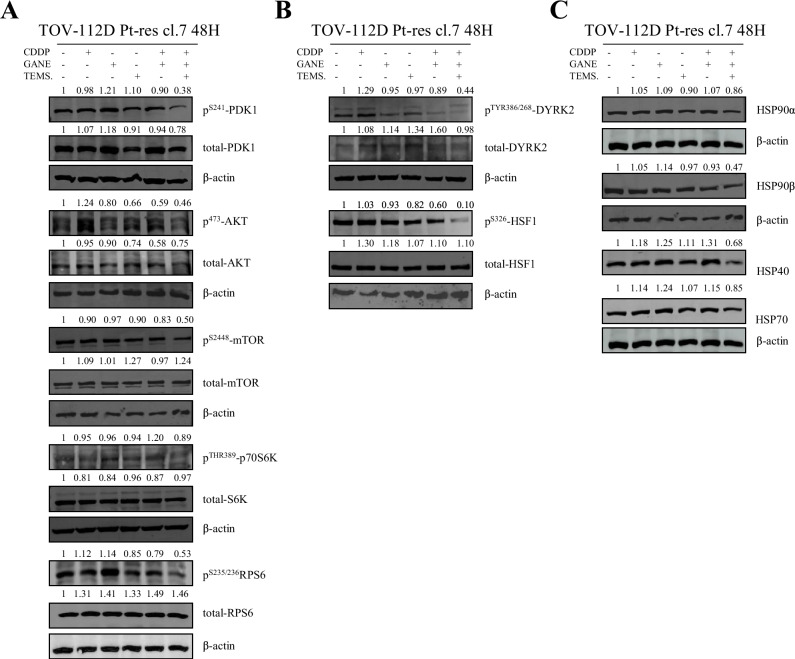
Effect of the triple combination on the main investigated pathways in Platinum-resistant EOC cells. Western blot analysis, performed after 48 h of treatment, of the main proteins (indicated in the figure) involved in mTOR mediated signaling pathway (**A**), HSF1 dependent transactivation (**B**) and chaperone complex (**C**) in TOV-112D Pt-res cl. 7 cells untreated or treated with CDDP, ganetespib, temsirolimus, and their combination at IC_50_^96h^ doses of parental cells. β-actin expression serves as loading control. Western blot quantification was performed by ImageJ software.

Mechanistically, we showed by western blot that the triple combination decreased the expression and the activation of the main proteins involved in mTOR signaling pathway, HSF1 transactivation and chaperone complex in TOV-112D Pt-res cl. 7 cells (Fig. 6A–C). These data were also confirmed in A549 CPr model, supporting the possibility that the same mechanism is present in different tumor types (Supplementary Fig. 8A–C). Notably, phosphoprotein analysis at 24 h in TOV-112D Pt-res cl.7 and A549 CPr cells revealed an early down-modulation, indicating that the triple combination rapidly interferes with key signaling cascades and exerts its effects already at early time points (Supplementary Fig. 9A, B), which become further pronounced at 48 h (Fig. 5A, B and Supplementary Fig. 8A, B).

### In vivo synergistic antitumor effect of ganetespib, temsirolimus and CDDP combination

**Fig. 7:**
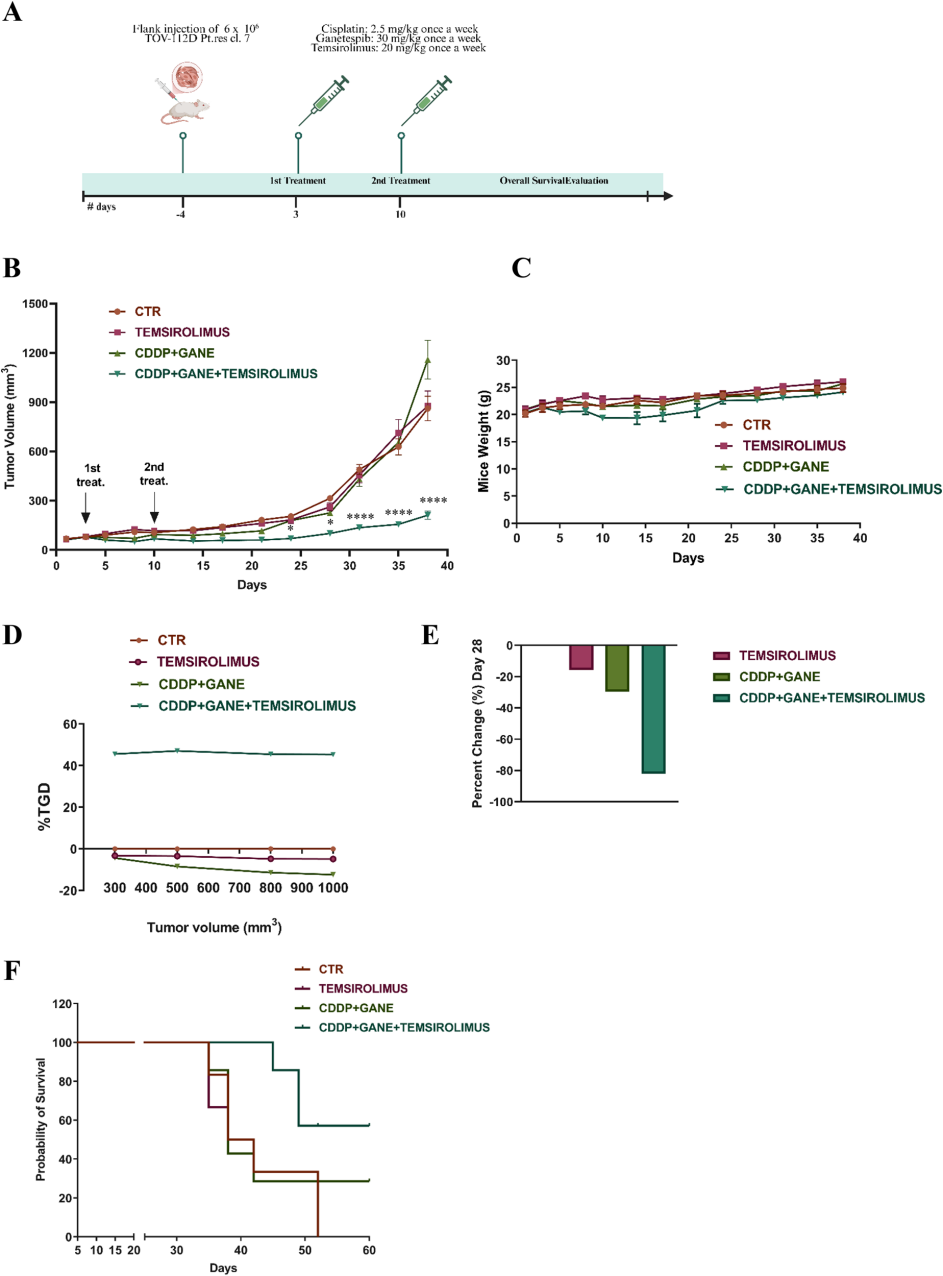
Potentiation of CDDP antitumor effect induced by ganetespib and temsirolimus in vivo Platinum-resistant TOV-112D xenograft model. **A** Schematic of the in vivo xenograft experiment, including timeline and agent concentration. TOV-112D Pt-res cl.7 cells (6 × 10^6^) were s.c. injected into NSG mice as described in Materials and Methods. When tumors were established, mice were treated once a week for two weeks with vehicles (CTR) or temsirolimus (20 mg kg^−1^ i.p.) or CDDP (2.5 mg kg^−1^ i.p.) and ganetespib (GANE; 30 mg kg^−1^ i.p.) or triple CDDP/ganetespib/temsirolimus combination. **B** Relative tumor volume (TV) measured at prespecified time points (Means ± SEM). **C** Mice body weight as surrogate indicator of toxicity for in vivo experiment reported in A. Body weight was measured three times/week. **D** TGD, indicating the mean rate of tumor growth in the treatment groups relative to control untreated mice (see Materials and Methods). **E** Percent change in tumor volume average from first day of treatment (day 0) to day 28 for each treatment group compared to vehicles group. **F** Kaplan–Meier curves comparing the survival of single and combination groups of treatment. Statistically significant results calculated with one-way ANOVA test comparing ganetespib plus CDDP and ganetespib/temsirolimus plus CDDP are reported (*P < 0.05 and ****P < 0.0001).

To evaluate the relevance of in vitro results we used the CDDP/ganetespib/temsirolimus combination to treat NSG mice-bearing tumors formed by TOV-112D Pt-res cl.7 cells (Fig. 7A). When tumors reached ~100 mm^3^ of volume, mice were randomized in four treatment groups: control, temsirolimus, CDDP plus ganetespib and CDDP plus ganetespib and temsirolimus. Significantly, as shown in Fig. 6, the triple combination treatment almost completely block tumor growth compared with the other experimental groups (Fig. 7B). We observed a modest decrease of mice body weight in the triple combination treatment; however, it was less than 10% and was not paralleled by other signs of acute or delayed toxicity (Fig. 7C). The synergistic effect of the CDDP/ganetespib/temsirolimus combination treatment was confirmed by the resulting tumor growth delay (TGD) that reached a peak of about 45% indicating that the mean rate of tumor growth was more than four-fold higher in the controls or the treated groups, than the combination setting (Fig. 7D). Moreover, by calculating the percent change in tumor volume from the time of initial treatment (day 3) to day 28, we demonstrated that temsirolimus, CDDP plus ganetespib and the triple combination treatment reduced the tumor burden by about 15, 30, and 85%, respectively (Fig. 7E). The reduced tumor growth also translated in a significant improvement of mice survival in the triple combination group, as demonstrated by Kaplan–Meier plot reported in Fig. 7F.

Finally, we confirmed the synergistic interaction of the proposed combination in A549 CPr cells xenograft model in athymic mice confirming that the triple combination lead to a reduction of the tumor volume compared with controls or other treated groups and a TGD that reached a peak of more than 100% (Supplementary Fig. 10A–C).

Overall our data demonstrated the efficacy and feasibility of CDDP plus ganetespib and temsirolimus combination in potentiating the antitumor effect of CDDP and to revert Pt-resistance in vivo in different tumor models.

## Discussion

Platinum (Pt)-based compounds are among the most commonly used chemotherapeutic agents to treat a wide variety of tumors and since their introduction into clinical practice, numerous omics-based studies have been conducted to address the clinically significant challenge of Pt-resistance that critically limits their efficacy [32, 33].

However, many of these studies have suffered from a major limitation due to the imprecise correlation between gene and protein expression, resulting in difficulty in identifying new effective therapeutic targets to overcome resistance [34]. Conversely, comprehensive analyses of the global proteome and phosphoproteome could provide new insights into the molecular mechanisms of chemoresistance and contribute to the identification of new biomarkers or therapeutic targets [35, 36]. Indeed, in the present study, by using a quantitative phosphoproteomics-based approach coupled with database analysis and bioinformatics tools, we identified the mammalian target of rapamycin (mTOR) and Heat Shock Factor 1 (HSF1)-activated multichaperone complex (HSP90, HSP70, and HSP40) as possible activated pathways by which cells acquire Pt chemoresistance. Notably, we validated the activation and the up-regulation of the main identified proteins involved in the pathways in both EOC and NSCLC Pt-res models.

The present study followed our experiences demonstrating, in line with others, that the multifunctional molecular chaperone HSP90, represents a central therapeutic hub in Pt-res EOC and NSCLC [15, 16]. Although we have clearly demonstrated the ability of the HSP90 clinically active inhibitor ganetesbip to synergize with CDDP in Pt-res EOC in vitro and ex vivo preclinical models, the antitumor effect of the combination in in vivo models, was less effective than expected [15]. HSP90 by regulating the post-translational stability and function of a distinct but diverse set of “client” proteins, known to be critically involved in oncogenesis is an appealing target for cancer therapy. High expression of HSP90 is associated with tumor growth and, inhibiting HSP90 activity has proven to disrupt multiple signal transduction pathways that are important for tumor development and survival [37]. Despite the extensive evaluation of HSP90 inhibitors (HSP90i) in clinical trials, none have received FDA approval, largely due to drug-related toxicity or therapy resistance as single agents. Notably, the two recently completed no-proft clinical studies investigating in ovarian cancer patients the combination of ganetespib in combination with either paclitaxel in Pt-resistance setting [27, 38] or with carboplatin followed by maintenance treatment with the PARP inhibitor Niraparib, in Pt-sensitive setting [39], have both shown no improved outcome, suggesting novel strategies to enhance the antitumor therapeutic response of this class of agents [40]. In this context, our work demonstrating the synergy between HSP90 and mTOR inhibition, might indicate a novel clinical possibility based on a solid molecular rationale.

The dysregulation of mTOR has been implicated in tumorigenesis, metastasis, and drug resistance across various human cancers [22, 41]. HSF1 is a master regulator responsible for inducing the expression of heat shock proteins (HSPs) and PI3K/AKT/mTOR oncogenic pathway activation [21].

Given the pivotal role of mTOR in coordinating various signaling pathways, it has been proposed as an attractive therapeutic target in cancer treatment [42]. The mTOR inhibitors function as antiproliferative drugs; however, as single agents, they have demonstrated limited efficacy in tumor eradication due to the activation of compensatory survival pathways. Nonetheless, mTOR inhibition has been shown to enhance the efficacy of various chemotherapeutic agents, including CDDP, carboplatin, paclitaxel, and doxorubicin, across different tumor types [43, 44]. In line with this, temsirolimus, a potent and selective inhibitor of mTOR, was approved by the European Medicines Agency for the treatment of relapsed/refractory Non-Hodgkin’s Lymphoma (Mantle Cell Lymphoma) [45] and by US Food and Drug Administration for poor prognosis metastatic renal cell carcinoma (mRCC) [46].

A phase II trial evaluating temsirolimus in combination with carboplatin and paclitaxel followed by temsirolimus consolidation in women with newly diagnosed clear cell ovarian cancer (CCOC), although well tolerated did not statistically significantly increase PFS at 12 months compared to historical controls [47]. Similarly, in women with Pt-refractory/resistant ovarian cancer or advanced/recurrent endometrial carcinoma, temsirolimus did not meet the predefined efficacy criteria, and few patients had long lasting disease stabilizations [48]. Conversely, based on our phosphoproteomics findings, here we demonstrated that triple combination of ganetespib and temsirolimus plus CDDP significantly enhanced the response to CDDP both in vitro and in vivo in Pt-resistant EOC and NSCLC xenograft models compared to single or double treatments. These data were also confirmed in vitro in HNSCC models. To our knowledge this is the first study evaluating the combination of an HSP90i such as ganetespib and temsirolimus in cancer models and the first to show the reversion of Pt-resistance by this original combination. Interestingly, mechanistically, this effect is linked to increased pro-apoptotic signaling and DNA damage, suggesting that targeting both mTOR and HSP90 disrupts critical survival pathways in Pt-res cells. In this regard, it has been demonstrated that mTOR inhibition activates autophagy to mediate the degradation of anti-apoptotic B cell lymphoma 2 (Bcl-2) protein to induce apoptosis [49]. Moreover, mTOR plays also a crucial role in the cellular response to CDDP by regulating apoptosis. In detail, a combination of the mTOR inhibitor rapamycin and the PI3K inhibitor LY294002 with CDDP has been shown to increase apoptosis and inhibit tumor growth in melanoma, correlating with the downregulation the anti-apoptotic protein Mcl-1 [50]. We also demonstrated that the triple combination treatment impaired proteins involved in mTOR signaling and the HSF1-activated multichaperone complex pathway, suggesting their potential association with off-target Pt-resistance mechanisms, along with other well-known pathways including autophagy, epigenetic changes, cancer stem cells (CSCs), epithelial-mesenchymal transition (EMT), and the heat shock response (HSR) [51]. Overall, our data support the possibility that the multimodal mechanisms of Pt-resistance acquisition in different cancer cellular models, which differ in histology and genetic backgrounds, are commonly dependent on the addiction, at least in part, to HSP90 and mTOR hub function.

In line with our data, in recent years, it has been proven that combining Hsp90 and mTOR inhibitors or dual HSP90-mTOR inhibitors could produce synergistic anti-cancer activity in various tumor such as cholangiocarcinoma, breast carcinoma, melanoma, and bladder cancer [52].

Although further studies should focus on better elucidating the molecular mechanisms underlying the synergistic antitumor effect of Hsp90 and mTOR inhibitors plus CDDP, our data provided a rationale to clinically explore this combination in CDDP refractory/resistant cancer patients. This combination treatment could have higher antitumor effects tested in patient stratified/selected, based on the evaluation of biomarkers within mTOR signaling and HSF1-HSP90 regulatory pathway, as suggested by our molecular findings.

## Materials and methods

### Cell culture conditions and cisplatin-resistant cell selection

TOV-112D (CRL-11731), NSCLC cell line A549 (CCL-185) and Cal33 (CVCL-1108) were purchased from ATCC (Manassas, VA, USA). CAL27 (CRL-2095) cell line was kindly provided by Dr. J. L. Fishel (Centre Lacassagne, Nice, France).

OVCAR-8 cells were purchased from National Cancer Institute Developmental Therapeutics Program Tumor Repository (Frederick, Maryland, USA). The green fluorescent protein + /luciferase+ (GFP + /Luc + ) Cal27 cell line were obtained by lentiviral infection as described previously [53].

TOV-112D CDDP-resistant cancer cells (referred as TOV-112D Pt-res cl-7), OVCAR 8 CDDP-resistant cancer cells (referred as OVCAR 8 Pt-res cl-2) and A549 CDDP-resistant cancer cells (CPr-A549) were generated as previously described [16, 18–20]. More details are described in Supplementary materials and methods. A549 heat-shock protein 90 alpha isoform (HSP90α) knockout cells were generated through the CRISPR-Cas9 system as reported previously [54]. The generated cell clones (A549 CPr KO#1, KO#3, KO#4) were analyzed by western blot and sequencing to verify knockout of HSP90α.

Patient-derived primary cells were described in [55, 56] and were obtained from patients who gave their informed consent, under protocols approved on 10.07.2019 by the Ethics Committee (OutCoME protocol - CRO-2019-53 approval CEUR 2019-Sper-084). The experiments were conformed to the principles set out in the WMA Declaration of Helsinki and the Department of Health Services Belmont Report. Patient data were pseudonymized and annotated in a prospective database. Primary cells were maintained in OCMI medium (M199 and Ham’s 1:1) supplemented with 2% FBS, EGF (10 ngmL^−1^), hydrocortisone (500 ngmL^−1^), cholera toxin (25 ngmL^−1^), and insulin (20 µMmL^−1^).

All cell lines were regularly inspected for mycoplasma. The cells have been authenticated with short tandem repeat profile generated by LGC Standards.

### Label free MS-based phosphoproteomics quantitation

In-solution digestion and phosphopeptide enrichment was performed. Purification of phosphopeptides was performed. Briefly, tryptic peptides were diluted six-fold in 80% (v/v) ACN, 6% (v/v) TFA (loading solution) and applied to TiO_2_ beads (about 50 μl slurry) pre-equilibrated in loading buffer and incubated for 15 min a room temperature in a Thermo-mixer R at a speed of 300 rpm. The samples were then washed three times with 50 μl of 80% ACN, 0.1% TFA (wash solution). Finally phosphopeptides were eluted with 100 μl of elution solution 1 (20 μl of 25% ammonium hydroxide solution [pH ≥ 10.5] + 980 μl H_2_O) followed by 100 μl of elution solution 2 (30% ACN). Both eluted 1 and 2 were dried in vacuum system (Concentrator 5301, Eppendorf, Milan, Italy). Samples were desalting by stage Tip C18 (Millipore, Merck KGaA, Munich, Germany) and lyophilized again. Desalted fractions were resuspended in 20 μl (approximately 5 μg of peptides) of 0.1% TFA and injected into Dionex UltiMate 3000 Rapid Separation LC nano system (Thermo Fischer Scientific, CA, USA) coupled with an AmaZon ETD mass spectrometer (Bruker Daltonics, Bremen, Germany). More details are described in Supplementary materials and methods.

### Phosphoproteomics quantification analysis

Progenesis QI for proteomics v. 4.2 (Non-linear Dynamics, Newcastle, England) was used as label-free quantification platform as previously described [57, 58]. The details are described in Supplementary materials and methods. Technical variability of each peptide/protein was estimated among replicates from the pooled sample by calculating Pearson correlation coefficients using the Perseus (v. 1.6.6.0) [59].

### Bioinformatic analysis

KEGG pathway, Functional Annotation Clustering and Gene Ontology Biological Process (GO-BP) analyses were performed using the Database for Annotation, Visualization and Integrated Discovery (DAVID) v6.8 (https://david.ncifcrf.gov/) using as background the *Homo Sapiens* proteome [60]. Enrichment of GO terms was considered statistically significant when corrected for multiple testing by the Benjamini-Hochberg method with adjusted *p* < 0.05. Pearson correlation analysis and volcano plot were calculated using the Perseus (v. 1.6.6.0) as reported by Tyanova et al. [59]. G:Profiler (version e94_eg41_p11) was performed as follows: Homo Sapiens was chosen as organism, GO analyses (GO molecular function (GO: MF) and GO cellular component (GO: CC), were carried out sequentially. The biological pathways were evaluated by Reactome (REAC) and WikiPathways (WP) databases. The statistical domain scope was used only for annotated genes. The significance threshold was the g:SCS threshold. The user threshold was *p* < 0.05 [61].

### Immunoblotting

Protein extraction after 48 h of cell culture and immunoblotting was performed as previously described [15]. Western blots were quantified using IMAGEJ software (Rasband, W.S., U.S., National Institutes of Health, Bethesda, MD, USA). For primary and secondary antibodies see Supplementary materials and methods.

### Cell proliferation assay and drug combination studies

cis-Diammine Pt (II) dichloride (CDDP) was provided by Sigma-Aldrich (St. Louis, MO, USA); Ganetespib was provided by MedChem Express (Sollentuna, Sweden) and stock solutions were prepared in DMSO. Temsirolimus (Torisel®) was provided by Pfizer. Cell viability and combination studies were measured using SRB assays after 96 h of treatment in 96-well plates, as described previously [15, 62].

### Microtissue formation assay

Ovarian cancer cell lines TOV-112D and TOV112D Pt-res cl.7 (800 cells) and Head and Neck Cal27-GFP^+^/Luc^+^ cancer cells (500 cells) were cultured as microtissues by the ultra-low attachment (ULA) System (PerkinElmer, Waltham, Massachusetts, USA). The ovarian cancer cells were marked using a green fluorescent probe-cell traker (Thermo Fisher Scientific, Waltham, Massachusetts, USA). The 3D microtissue model for the EOC cell lines was obtained using mouse fibroblast cells NIH/3T3 as scaffold in a ratio of 3:1 as described in literature and untreated or treated with drugs, for 96 h with CDDP, ganetespib and temsirolimus alone or in combination at IC_50_^96h^ doses of parental cells. 3D microtissues were maintained in the incubator and photographed by Opera Phenix microscope (PerkinHelmer, Waltham, Massachusetts, USA) air objective magnification 5X and scored by Cell Titer-Glo® 3D Cell Viability Assay (Promega, Madison, Wisconsin, USA) by using a Multimode Reader Cytation 5 (Biotek, Santa Clara, California, USA).

### In vivo xenograft studies

All studies have been performed in compliance with institutional guidelines and regulations that cover all scientific procedures involving the use of live animals (Directive 2010/ 63/EU; Italian Legislative Decree DLGS 26/2014).

Female NOD scid gamma (NSG) mice (4–6 weeks old) and female athymic (nude) mice (4–6 weeks old) were acquired from Charles River Laboratories (Charles River, Wilmington, MA, USA) and used for TOV-112D Pt-res cl.7 and A549 CPr xenograft model, respectively. Mice were acclimatized in the Animal Care Facility of ‘Fondazione G. Pascale’-IRCCS/Laboratori di Mercogliano-CROM and housed in a 12 h light: 12 h dark cycle in a controlled room temperature of 22 ± 2 °C and fed ad libitum. After 3 days, TOV-112D Pt-res cl.7 cells (6 × 10^6^) diluted in 200 mL [PBS/Matrigel GF (Becton Dickinson, Franklin Lakes, NJ, USA) 1/1] were injected subcutaneously (s.c) in flank regions of the mice. When the tumors became palpable, the mice were randomized into four experimental groups (*n* = 7). Intraperitoneal (i.p.) treatments were performed with temsirolimus (20 mg kg^−1^, dissolved in 10% DMSO + 40% poly (ethylene glycol) + 5% TWEEN80 + 45% physiological solution) or CDDP (2.5 mg kg^−1^, dissolved in PBS) and ganetespib (30 mg kg^−1^, dissolved in 10% DMSO + 40% poly (ethylene glycol) + 5% TWEEN80 + 45% physiological solution) or triple CDDP/ganetespib/temsirolimus combination, or vehicles, once a week.

A549 CPr cells (5 × 10^6^) were injected s.c in flank region of the nude mice. When the tumors became palpable, the mice were randomized into eight experimental groups (*n* = 7). I.p. treatments were performed with CDDP (2.5 mg kg^−1^) and/or ganetespib (75 mg kg^−1^) and/or temsirolimus (20 mg kg^−1^) or vehicles, once a week. Mice in the control groups were treated with both PBS and 10% DMSO + 40% poly (ethylene glycol) + 5% TWEEN80 + 45% physiological solution.

Sample size was based on estimations by power analysis with a level of significance of 0.05 and a power of 0.8 (G*Power version 3.1.9.7).

We took adequate steps to insure that animals did not suffer unnecessarily at any stage of the experiment described above.

Tumor volume (mm^3^), tumor growth delay (TGD) and the percent change in tumor volume from the time of initial treatment to the end of the study were evaluated as described before [63]. Overall survival were calculated considering as positive event in Kaplan-Meier curves the achievement of humane end point (Volumetric cut-off:1500 cm^3^; Weight cut-off: 20% reduction).

### Statistical analysis

The statistics of the shotgun proteomics experiment is performed by Progenesis QI for proteomics v. 4.2 and reported in the specific section.

Graphs and data analyses were carried out utilizing PRISM software (version 9, GraphPad, Inc., Ritme Informatique, Paris, France). Where the means of two data sets were compared, and significance was determined by a two-tailed Students *t* test or ANOVA, as indicated in each figure. Differences was considered significant at *p* < 0.05 (*p ≤ 0.05, **p ≤ 0.01, *** *p* ≤ 0.001, ****p ≤ 0.0001).

## Supplementary information

Supplementary Figures

Supplementary Figure legends

Supplementary Tables

Supplementary Materials and Methods

WB uncropped

## Data Availability

The raw data generated in this study are publicly available in Zenodo (10.5281/zenodo.16564396). All datasets are available from the corresponding author on reasonable request
