# Supplementary material for: Dual inhibition of mTOR and HSP90 enhances cisplatin efficacy and overcomes resistance in ovarian cancer
Source: Cell Death Dis. 2026 Mar 27;17(1):417. doi: 10.1038/s41419-026-08533-3 (PMC13149855; doi:10.1038/s41419-026-08533-3)
Supplement: Supplementary file 1 — Supplementary Figures [file 41419_2026_8533_MOESM1_ESM.pdf]

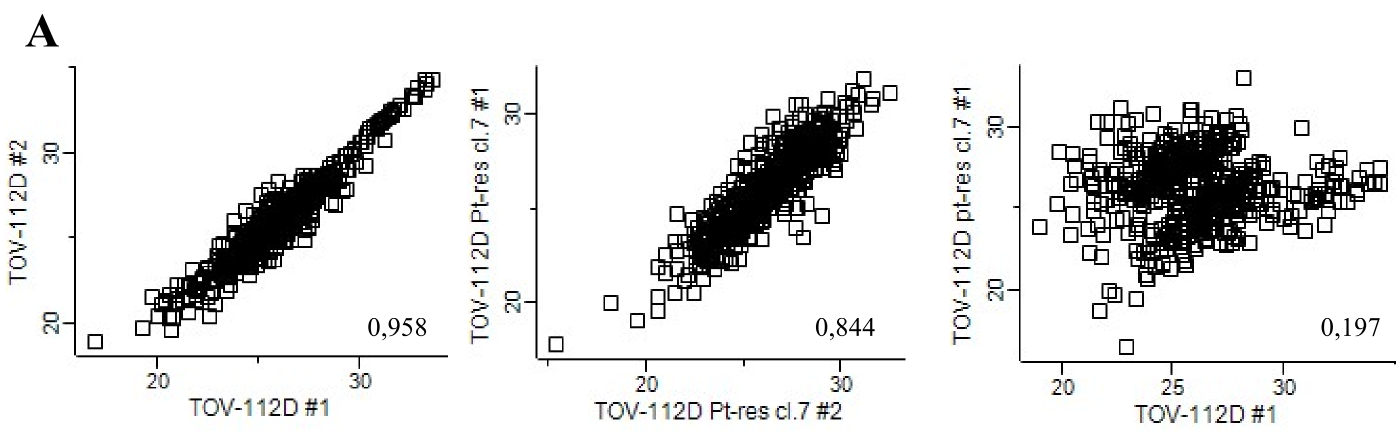

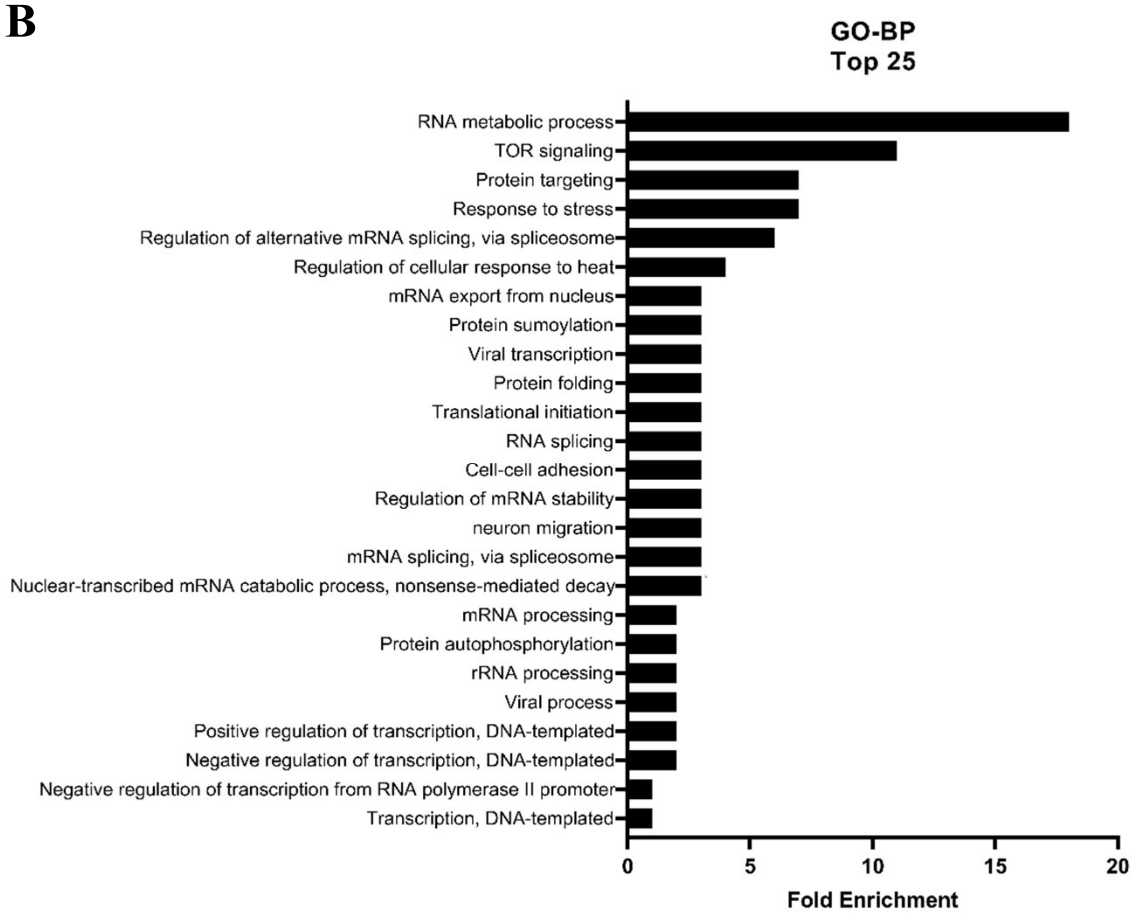

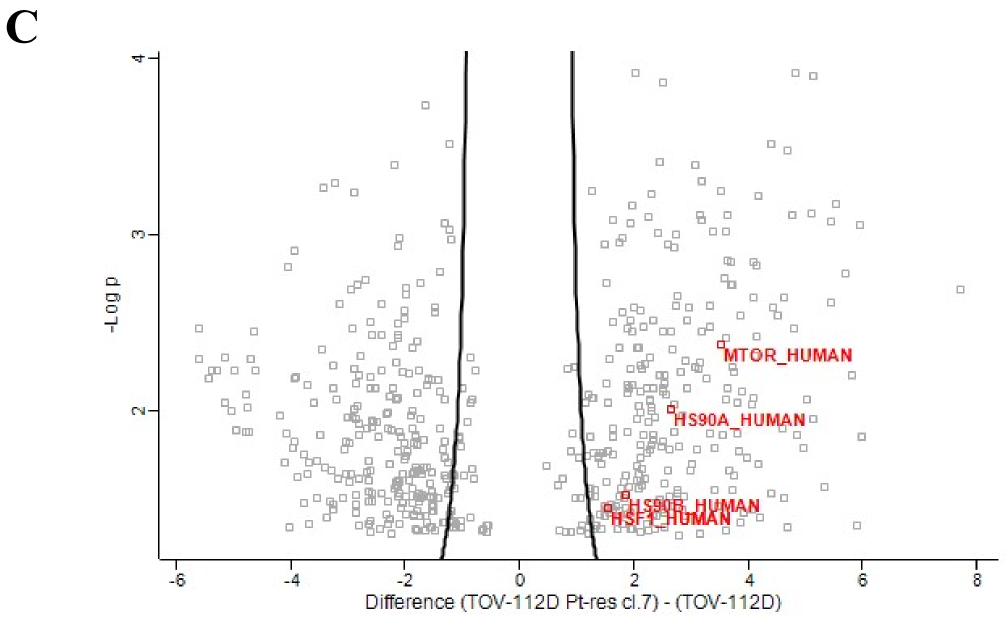

Suppl. Figure 1

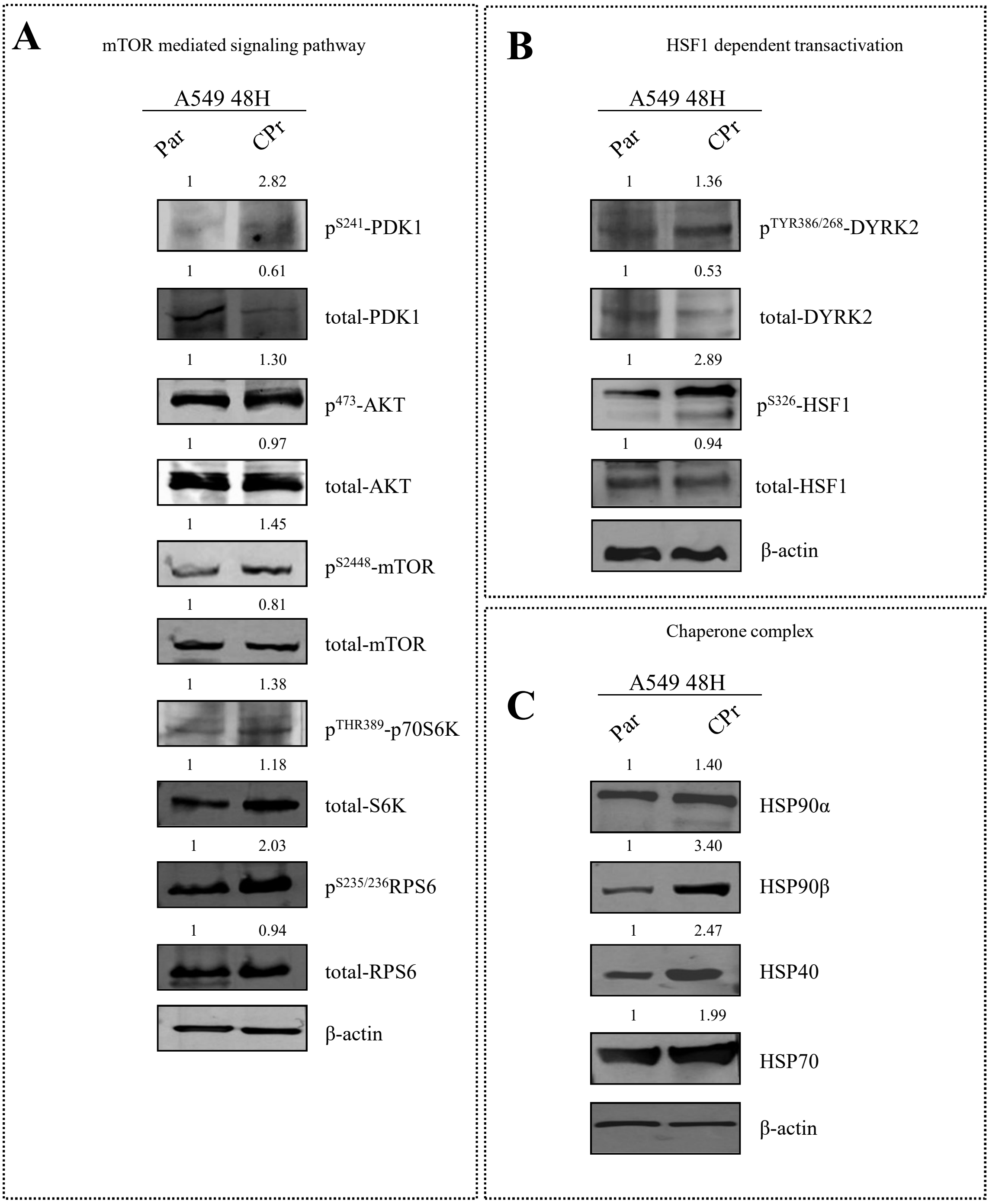

Suppl. Figure 2

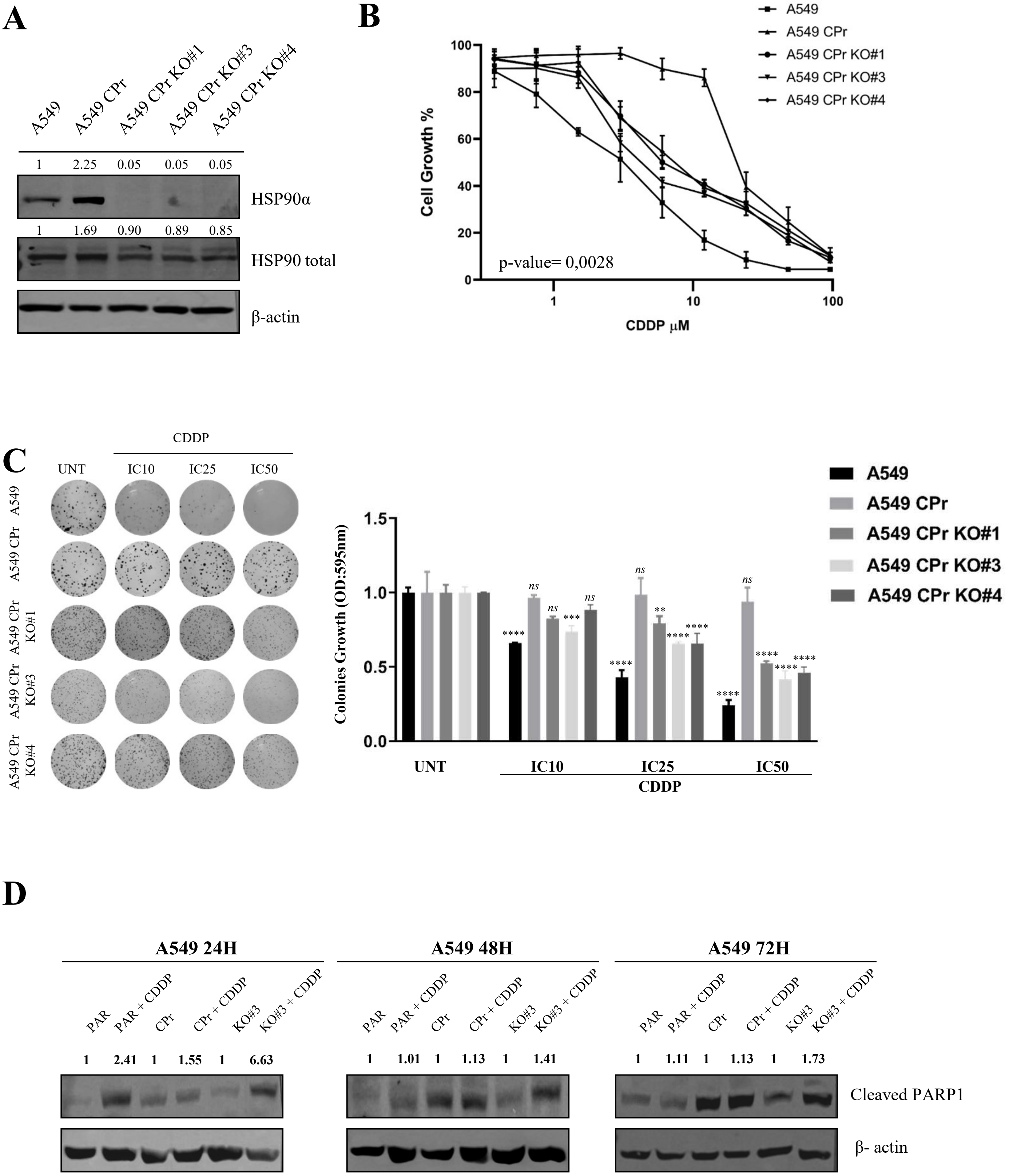

Suppl. Figure 3

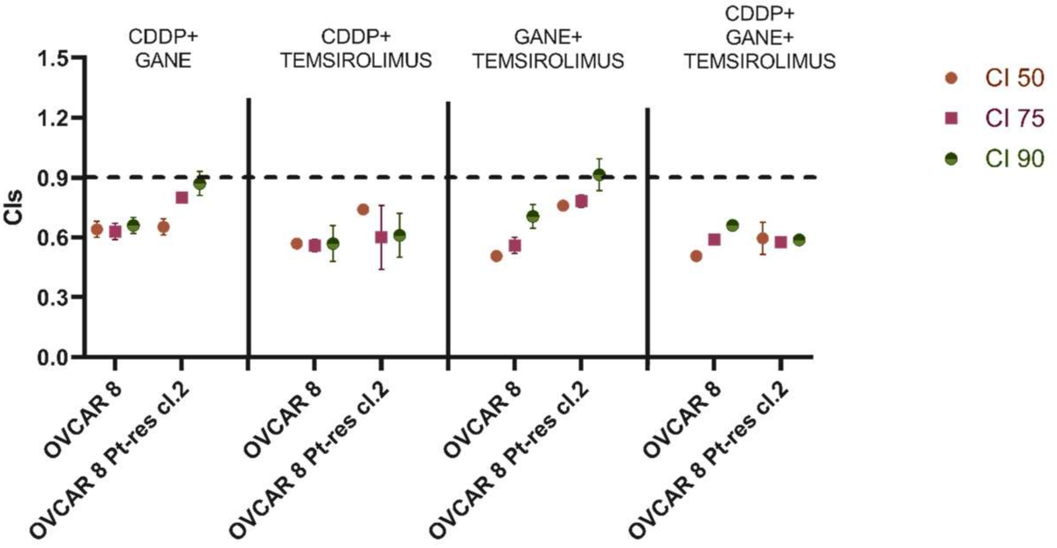

Suppl. Figure 4

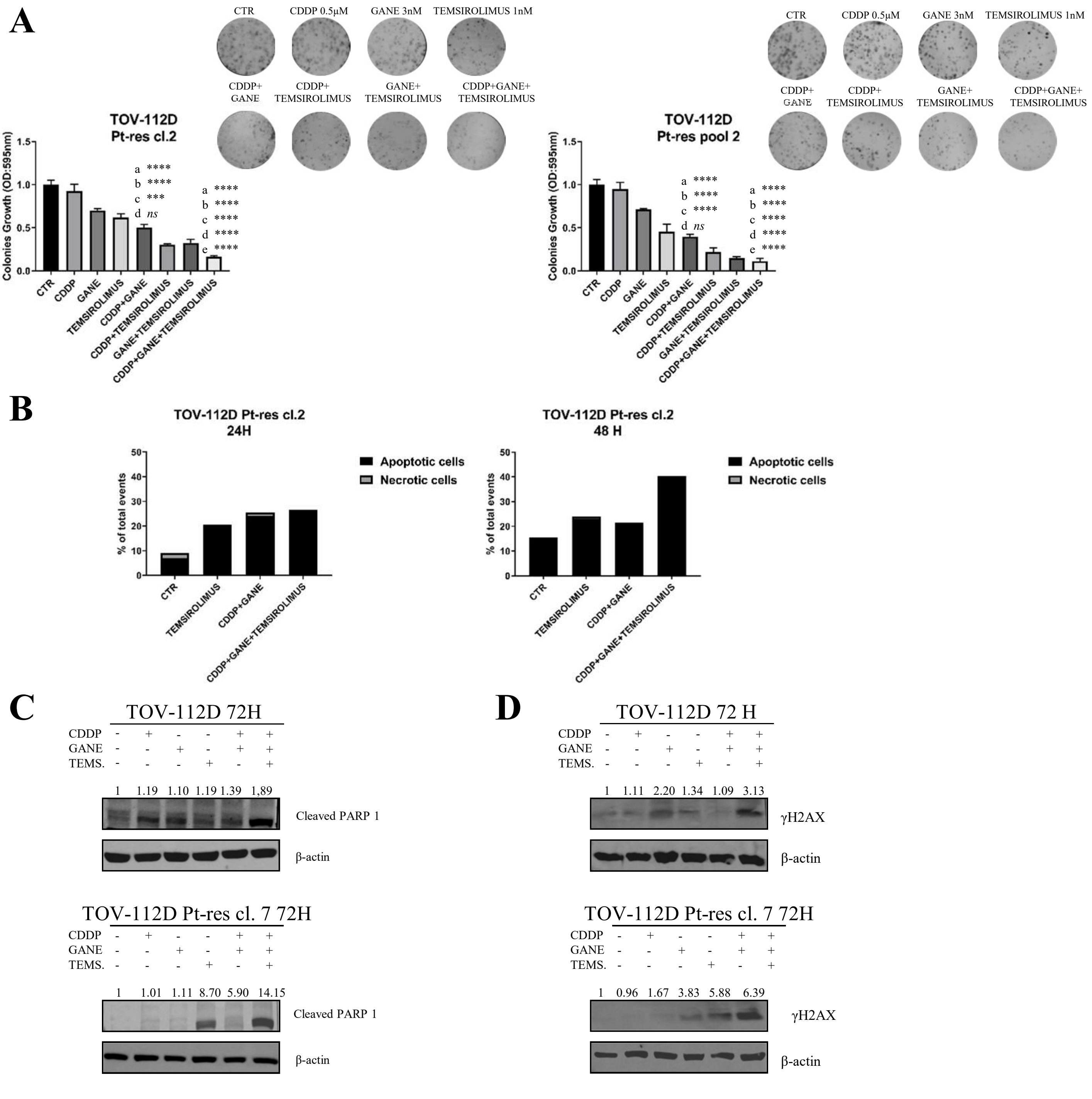

Suppl. Figure 5

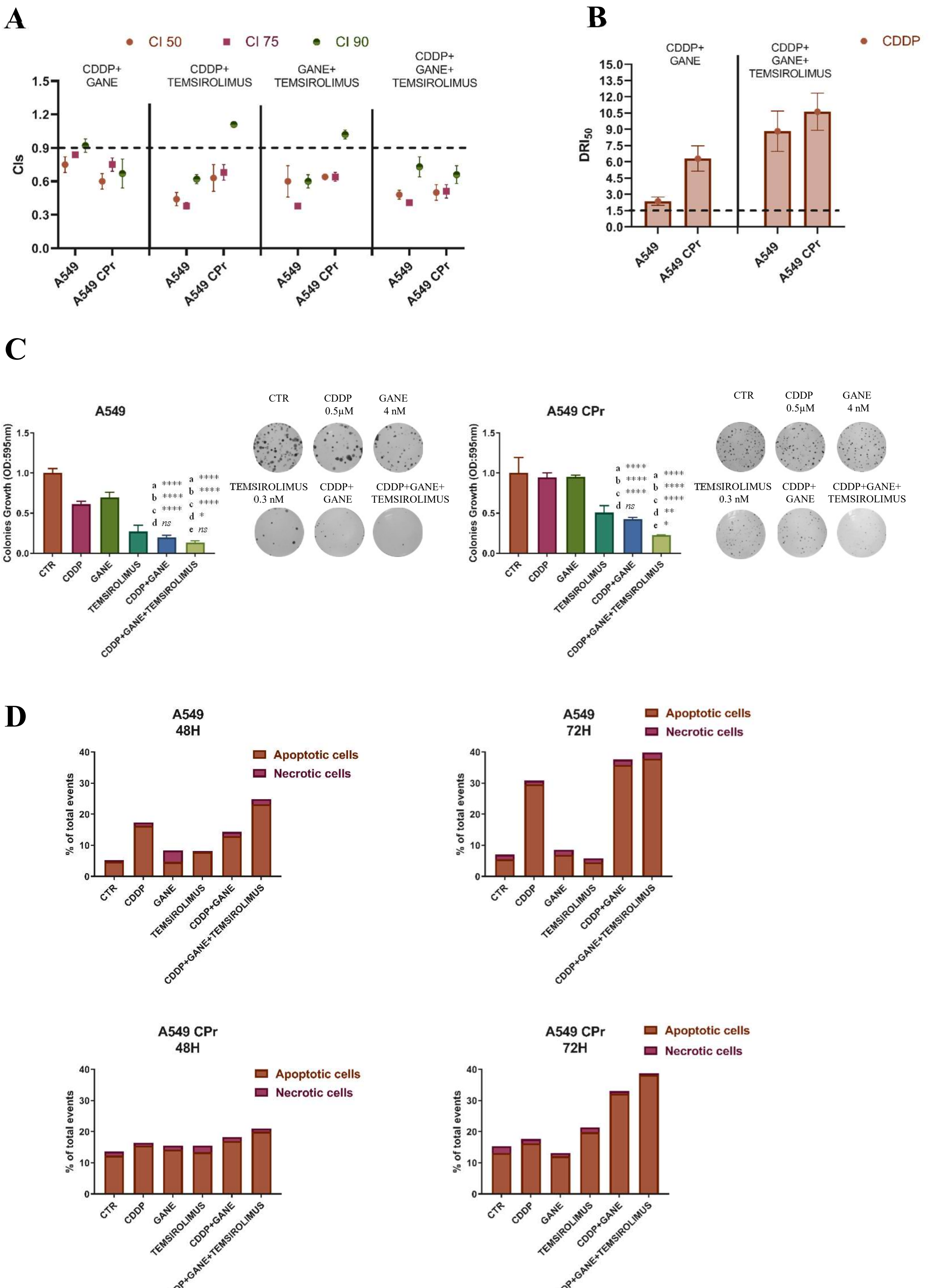

Suppl.Figure 6

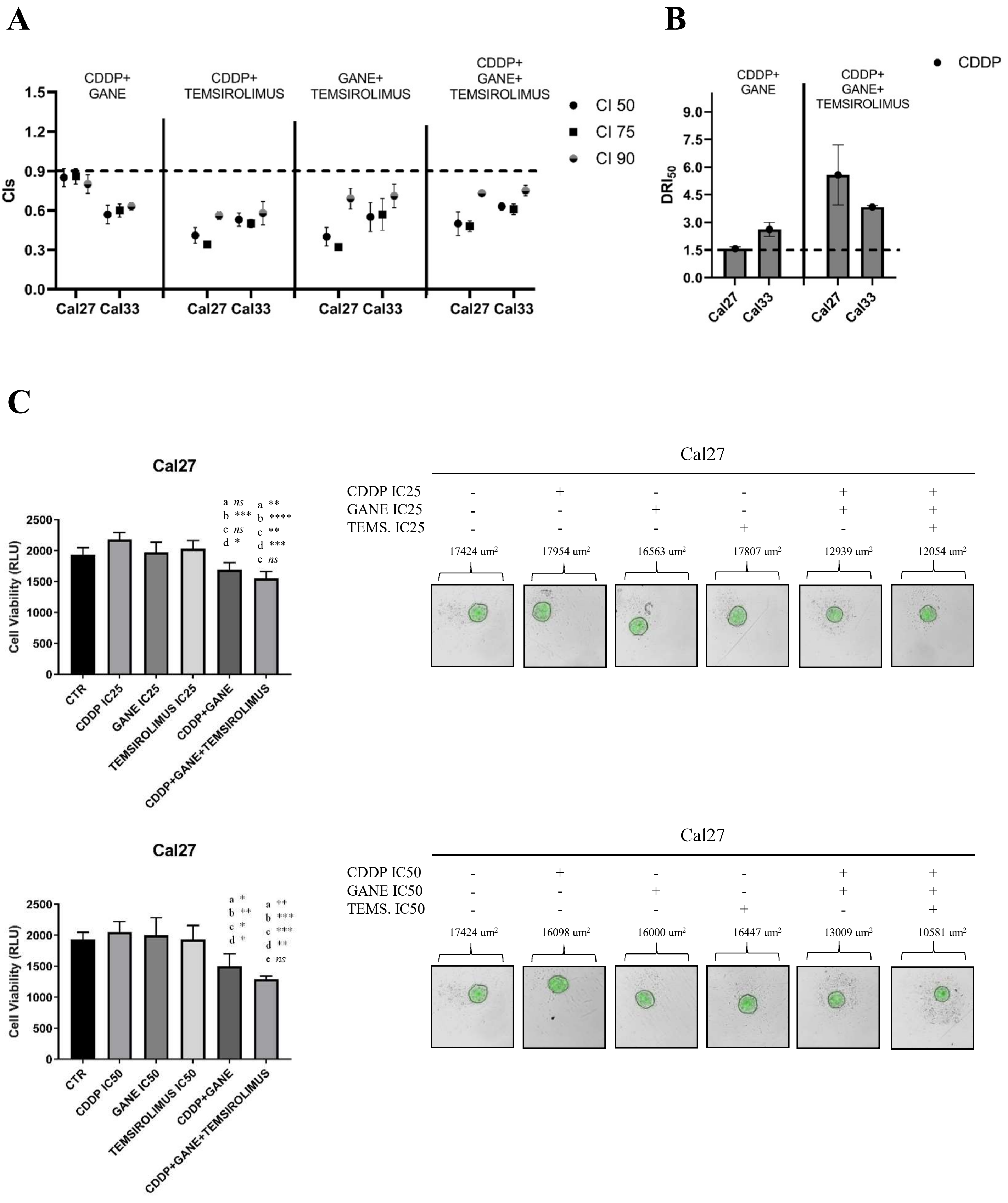

Suppl. Figure 7

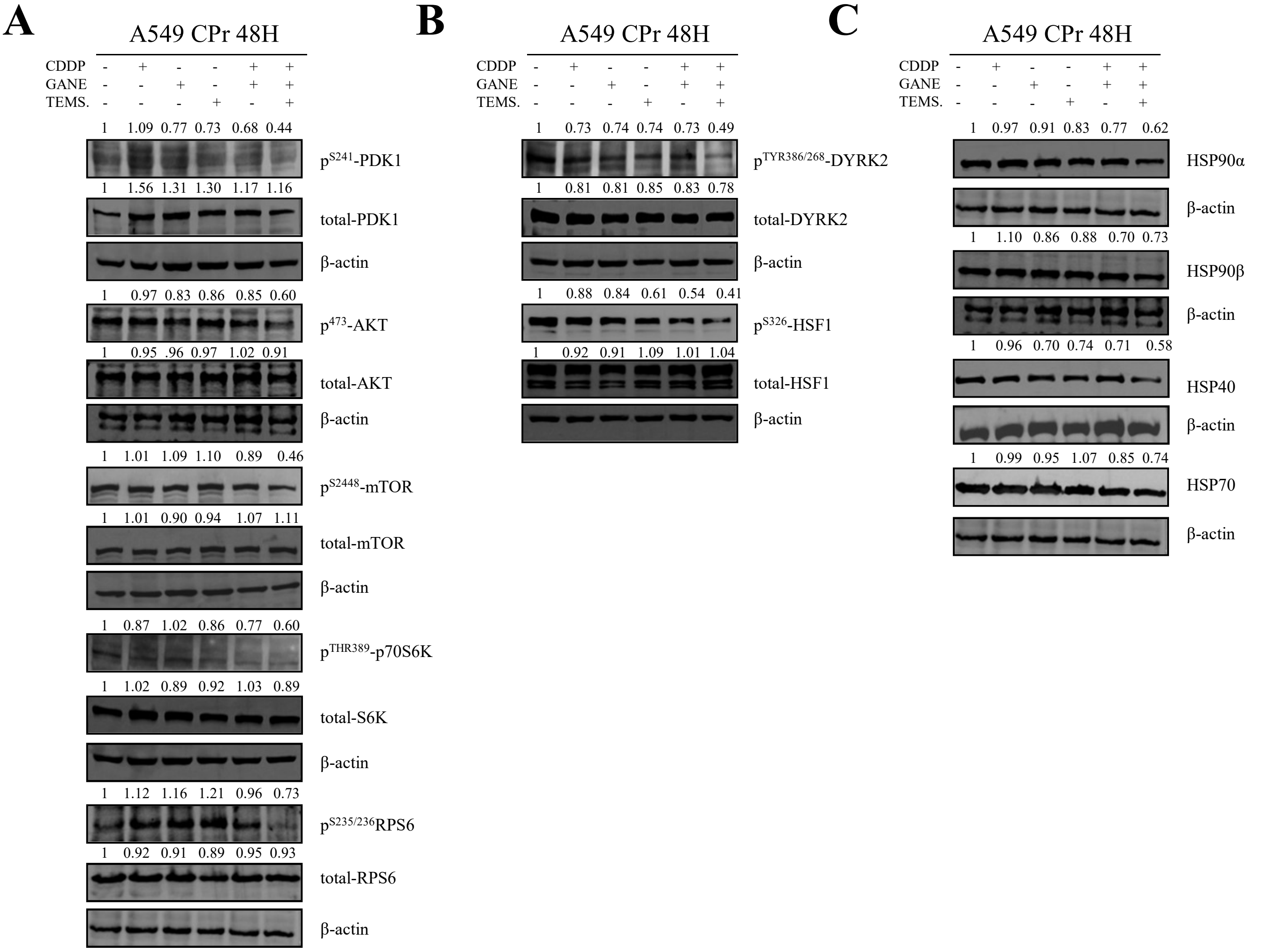

Suppl. Figure 8

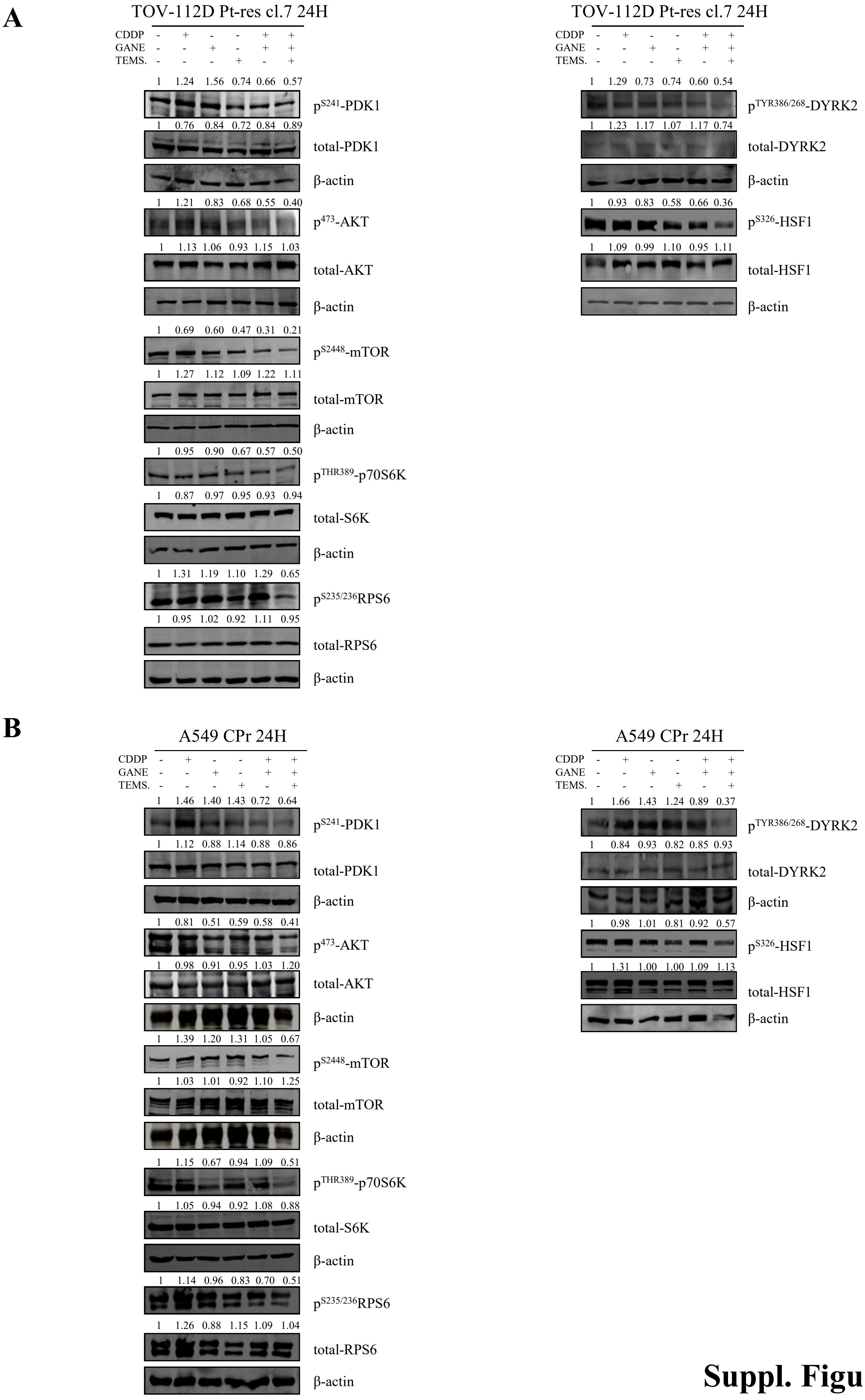

Suppl. Figure 9

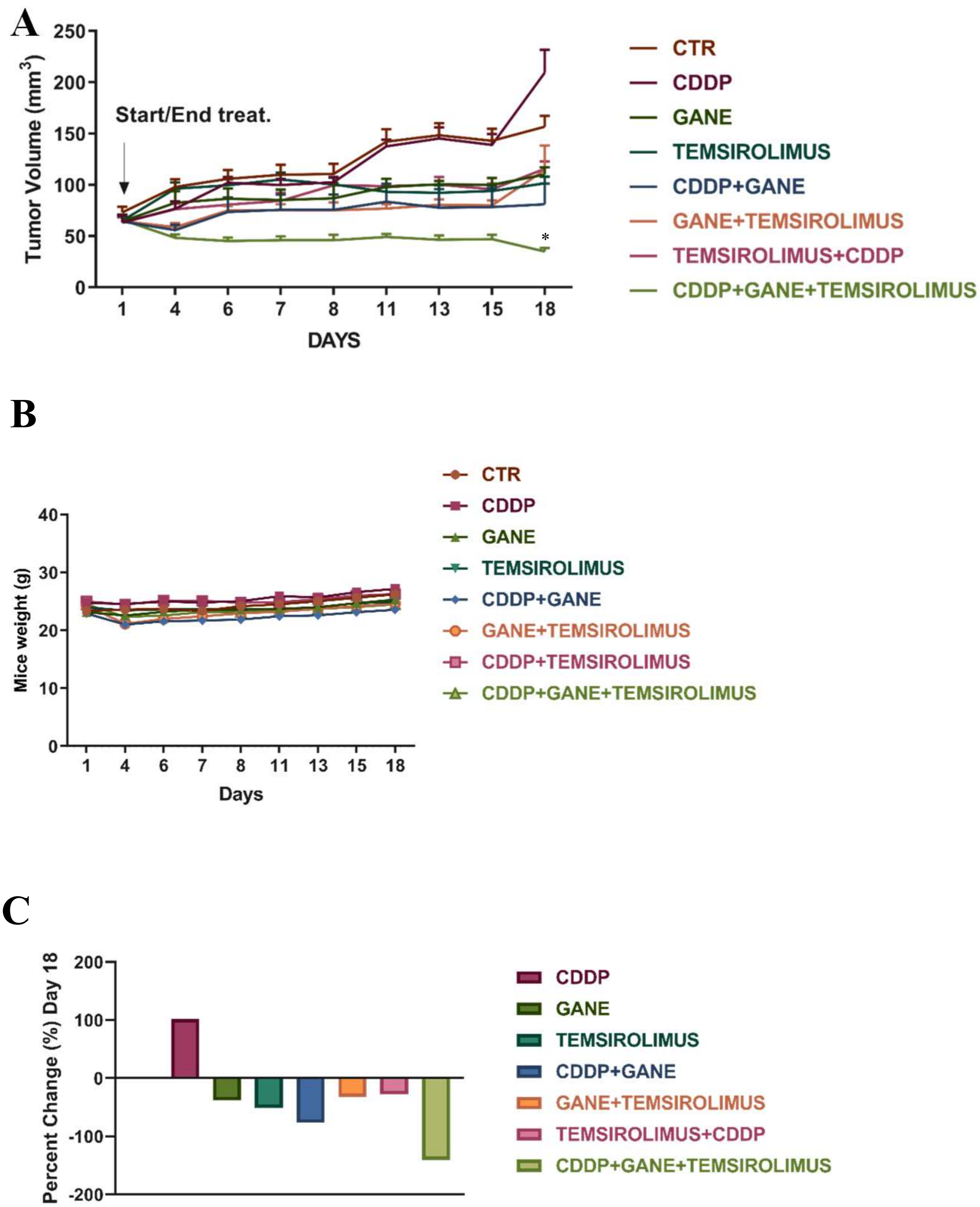

Suppl. Figure 10
