# Supplementary material for: Dual inhibition of mTOR and HSP90 enhances cisplatin efficacy and overcomes resistance in ovarian cancer
Source: Cell Death Dis. 2026 Mar 27;17(1):417. doi: 10.1038/s41419-026-08533-3 (PMC13149855; doi:10.1038/s41419-026-08533-3)
Supplement: Supplementary file 2 — Supplementary Figure legends [file 41419_2026_8533_MOESM2_ESM.pdf]

Supplementary Fig. S1. Label-free LC-MS MS-based phosphoproteomics quantification and bioinformatics analysis. A Scatter plot depicting the Pearson's correlation between the biological replicates. An absolute value of about 0.8-1 indicated a very good linear relationship. **B** Functional enrichment analysis of the significant proteins using DAVID software in terms of Gene Ontology (GO) Biological Processes (BP). The top-25 results are visualized. **C** Volcano plots of phosphoproteins abundance between TOV-112D and TOV-112D Pt-res cl.7. Depicted in the plots are the comparisons of log2 fold changes (LFC) *vs* p-values (Student's t-test between replicate measurements). Red dots: upregulated phosphoproteins (log2 fold change ≥ 1.5; p-value < 0.05).

Supplementary Fig. S2. Western blot validation experiments of the identified phosphoproteins in A549 cellular model. A Western blot analysis, performed after 48h of cell culture, showed an activation of mTOR mediated signalling pathway thorough the phosphorylation of p^{S241}-PDK1, p^{S473}-AKT, p^{S2448}-mTOR, p^{THR389}-p70S6K, $p^{S235/236}$RPS6 proteins in A549 CPr resistant cells *vs* A549 parental cells (Par). Densitometric analysis was done by ImageJ software and reported as ratio: phospho-protein/total protein/loading control (ß-actin). **B** HSF1 dependent transactivation was confirmed by western blot analysis performed after 48h of cell culture, showing an upregulation of $p^{TYR386/268}$-DYRK2 and p^{S326}-HSF1 proteins in A549 CPr resistant cells *vs* A549 parental cells (Par). Densitometric analysis was done by ImageJ software and reported as ratio: phospho-protein/total protein/loading control (ß-actin). **C** Expression of the proteins of chaperone complex (HSP90α, HSP90β, HSP40, HSP70) was evaluated by western blot experiment in A549 CPr resistant cells *vs*A549parental cells (Par) performed after 48h of cell culture. Densitometric analysis was done by ImageJ software and reported as ratio relative to the indicated loading control (ß-actin).

Supplementary Fig. S3. HSP90*α* knockout sensitizes Pt-res A549 CPr cells to CDDP treatment. A Western blot analysis evaluating the expression of HSP90α and HSP90 total in A549 parental, A549 CPr and HSP90α knockout CPr KO#1, CPr KO#3 and CPr KO#4 cells. β-Actin was used as loading control. Western blot quantification was performed by ImageJsoftware. B A549 parental, A549 CPr and HSP90α knockout CPr KO#1, CPr KO#3 and CPr KO#4 cells were treated for 96h with increasing concentrations of CDDP. Cell growth expressed as percentage of control was assessed by sulforhodamine B colorimetric assay (see Materials and methods). Statistically significant results are reported (P = 0.0028). C Clonogenic assay of A549 parental, A549 CPr and HSP90α knockout CPr KO#1, CPr KO#3 and CPr KO#4 cells treated with CDDP at the IC_{10}^{96h}, IC_{25}^{96h} and IC_{50}^{96h} doses for parental cells. Representative data of at least three independent experiments performed in triplicates. Statistically significant results (respect each untreated group) calculated with one-way ANOVA test are reported (*P < 0.05, **P < 0.01, ***P < 0.001, and ****P < 0.0001, ns, not statistically significant). D Western blot analysis of cleaved PARP1 expression in A549 parental, A549 CPr and HSP90α knockout CPr KO#3 cells, untreated or treated for 24, 48 or 72h with CDDP at IC_{50}^{96h} doses of parental cells.

Supplementary Fig. S4. Potentiation of CDDP antitumor effect induced by ganetespib and temsirolimus in parental and Pt-resistant OVCAR 8 cells. CI (combination index) values (mean ± SD from at least three separate experiments performed in quadruplicates) computed at 50% (CI50), 75% (CI75) and 90% (CI90) of cell kill by CalcuSyn software after 96h for OVCAR 8 and OVCAR 8 Pt-res cl.2 cells. The treatments are indicated in the figure. The combinations were considered synergistic when CIs were below 0.9, additive when CIs were between 0.9 and 1.1 or antagonism when Cis were more than 1.1.

Supplementary Fig. S5. Antiproliferative, proapoptotic and DNA damage effect induced by the triple combination treatment in Pt-resistant TOV-112D cells. A Synergistic inhibition of colony formationin TOV-112D Pt-res cl.2 and TOV-112D Pt-res pool 2 treated with CDDP, ganetespib, temsirolimus or their combination (simultaneous exposure) at the IC_{10}^{96h} doses for parental cells. Representative data of at least three independent experiments performed in triplicates. Statistically significant results calculated with one-way ANOVA test are reported (a indicates control group, b indicates CDDP-treated cells, and c indicates ganetespib-treated cells, d indicates temsirolimus-treated cells, e indicates CDDP plus ganetespib-treated cells *P < 0.05, **P < 0.01, ***P < 0.001 and

****P < 0.0001, ns, not statistically significant). **B** Apoptosis and necrosis evaluated by flow cytometry after Annexin V-FITC and propidium iodide staining in TOV-112D Pt-res cl. 2, untreated or treated for 24h or 48h, with temsirolimus, CDDP plus ganetespib and CDDP plus ganetespib and temsirolimus at IC_{50}^{96h} doses of parental cells. **C** Western blot analysis of cleaved PARP1in TOV-112D and TOV-112D Pt-res cl. 7 cells untreated or treated with CDDP, ganetespib, temsirolimus and their combination at IC_{50}^{96h} doses of parental cells at the time indicated above. β-actin expression serves as loading control. Western blot quantification was performed by ImageJ software. **C** Western blot analysis of γH2AX in TOV-112D and TOV-112D Pt-res cl. 7 cells untreated or treated with CDDP, ganetespib, temsirolimus and their combination at IC_{50}^{96h} doses of parental cells at the time indicated above. β-Actin expression serves as loading control. Western blot quantification was performed by ImageJ software.

Supplementary Fig. S6. Antiproliferative and proapoptotic effect induced by the triple combination treatment in A549 parental and A549 CPr resistant cells. A CI (combination index) values (mean ± SD from at least three separate experiments performed in quadruplicates) computed at 50% (CI50), 75% (CI75) and 90% (CI90) of cell kill by CalcuSyn software after 96h for A549 and A549 CPr cells. The treatments are indicated in the figure. The combinations were considered synergistic when CIs were below 0.9, additive when CIs were between 0.9 and 1.1 or antagonism when Cis were more than 1.1.**B** DRI (doses reduction index) values (mean ± SD) for CDDP from at least three separate experiments performed in quadruplicate) that represent the order of magnitude (fold) of dose reduction obtained for IC50 (DRI50) in combination setting compared with each drug alone in A549 and A549 CPr cells. **C** Synergistic inhibition of colony formation in A549 and A549 CPr cells treated with CDDP, ganetespib, temsirolimus or their combination (simultaneous exposure) at the IC_{10}^{96h} doses of parental cells. Representative data of at least three independent experiments performed in triplicates. Statistically significant results calculated with one-way ANOVA test are reported (a indicates control group, b indicates CDDP-treated cells, and c indicates ganetespib-treated cells, d indicates temsirolimus-treated cells, e indicates CDDP plus ganetespib-treated cells *P < 0.05, **P < 0.01, ***P < 0.001 and ****P < 0.0001, ns, not statistically significant). **D** Apoptosis and necrosis evaluated by flow cytometry after Annexin V-FITC and propidium iodide staining in A549 and A549 CPr cells, untreated or treated for 48h or 72h with CDDP, ganetespib, temsirolimus or their combination at IC_{50}^{96h} doses of parental cells.

Supplementary Fig. S7. Potentiation of CDDP antitumor effect induced by ganetespib and temsirolimus in Head and Neck cancer cells. A CI (combination index) values (mean ± SD from at least three separate experiments performed in quadruplicates) computed at 50% (CI50), 75% (CI75) and 90% (CI90) of cell kill by CalcuSyn software after 96h for Cal27 and Cal33 cells. The treatments are indicated in the figure. The combinations were considered synergistic when CIs were below 0.9, additive when CIs were between 0.9 and 1.1 or antagonism when Cis were more than 1.1.**B** DRI (doses reduction index) values (mean ± SD) for CDDP from at least three separate experiments performed in quadruplicate) that represent the order of magnitude (fold) of dose reduction obtained for IC50 (DRI50) in combination setting compared with each drug alone in Cal27 and Cal33 cells. **C** Synergistic inhibition of microtissues formation by CDDP, ganetespib, temsirolimus alone and in combination. Cal27-GFP^{+}/Luc^{+} cancer cells were plated in each well and after 24h treated withIC_{25}^{96h}or IC_{50}^{96h} doses. Representative images from Opera Phenix confocal microscopy. The graphics represent the number of viable cells in 3D cell culture based on quantitation of the ATP content. Results were obtained by a single experiment performed in triplicate (±SD). Statistically significant results calculated with one-way ANOVA test are reported (a indicates control group, b indicates CDDP-treated cells, and c indicates ganetespib-treated cells, d indicates temsirolimus-treated cells, e indicates CDDP plus ganetespib-treated cells *P < 0.05, **P < 0.01, ***P < 0.001 and ****P < 0.0001, ns, not statistically significant).

Supplementary Fig. S8. Effect of the triple combination on the main investigated pathways in A549 CPr cells. A-C Western blot analysis, performed after 48h of treatment, of the main proteins (indicated in the figure) involved in mTOR mediated signalling pathway (**A**), HSF1 dependent

transactivation (**B**) and chaperone complex (**C**) in A549 CPr cells untreated or treated with CDDP, ganetespib, temsirolimus and their combination at IC_{50}^{96h} doses of parental cells. β-actin expression serves as loading control. Western blot quantification was performed by ImageJ software.

Supplementary Fig. S9. Early effect of the triple combination on key phosphoproteins in TOV-112D Pt-res cl.7 and A549 CPr cells. Western blot analysis showing the main phosphoproteins (indicated in the figure) involved in the mTOR signaling pathway and HSF1-dependent transactivation in (A) TOV-112D Pt-res cl.7 and (B) A549 CPr cells, either untreated or treated for 24 hours with CDDP, Ganetespib, Temsirolimus, or their triple combination at IC_{50}^{96h} doses of the parental cells. β-actin was used as loading control. Protein quantification was performed using ImageJ software.

Supplementary Fig. S10. Potentiation of CDDP antitumor effect induced by ganetespib and temsirolimus *in vivo* Pt-resistant A549 xenograft model. A549 CPr cells (5×10^6) were s.c. injected into athymic mice as described in Materials and Methods. When tumors were established, mice were treated only once with vehicles (CTR) or CDDP (2.5 mg·kg−1 i.p.) or ganetespib (GANE; 75 mg·kg−1 i.p.) or temsirolimus (20 mg·kg−1 i.p.) or their combinations. **A** Relative TV measured at prespecified time points (Means ± SEM). **B** Mice body weight as surrogate indicator of toxicity for *in vivo* experiment reported in A. Body weight was measured three times/week. **C** Percent change in tumor volume average from first day of treatment (day 0) to the end of the study (day 18) for each treatment group compared to vehicles group. Statistically significant results calculated with one-way ANOVA test comparing ganetespib plus CDDP and ganetespib/temsirolimus plus CDDP are reported ($*P < 0.05$).
