# Supplementary material for: Dual inhibition of mTOR and HSP90 enhances cisplatin efficacy and overcomes resistance in ovarian cancer
Source: Cell Death Dis. 2026 Mar 27;17(1):417. doi: 10.1038/s41419-026-08533-3 (PMC13149855; doi:10.1038/s41419-026-08533-3)
Supplement: Supplementary file 3 — Supplementary Tables [file 41419_2026_8533_MOESM3_ESM.pdf]

Supplementary Table 1. Sensitivity of EOC and NSCLC cell lines to single agent treatments.

CELL LINES	CDDP<br>IC50 (μM)<br>96h ± SD	GANE<br>IC50 (nM)<br>96h ± SD	TEMS<br>IC50 (nM)<br>96h ± SD
**TOV-112D**	2.2 5± 0.09	13.10± 2.15	6.33 ± 1.55
**TOV-112D<br>Pt-res cl.7**	7.62 ±0.30	24.09± 2.24	32.73 ± 8.33
**OVCAR 8**	1.82± 0.50	7.53± 0.78	5.66±2.04
**OVCAR 8<br>Pt-res cl.2**	7.01±.1.49	12.94± 0.78	nd
**A549**	2.31 ± 0.27	7.9 1± 1.22	6.20 ± 0.79
**A549 CPr**	26.52 ± 4.45	13.28 ± 0.82	23.15 ± 4.22

Abbreviations: CDDP: Cisplatin; GANE: ganetespib; TEMS: Temsirolimus.

IC: Inhibitory Concentration. The IC_{50} values were computed at 96h of treatment (mean±SD) from at least three separate experiments performed in triplicates. Cell growth assessment was done by sulforhodamine B colorimetric assay (see Materials and Methods).

Supplementary Table 2. Combination index (CI) and dose reduction index (DRI) values for Cisplatin (CDDP), Ganetespib (GANE) and Temsirolimus (TEMS) combinations treatment accordingly to different treatment schedules in different EOC models.

Cell Lines	CDDP+GANE	CDDP+TEMS	GANE+TEMS	CDDP+GANE+TEMS
**TOV-112D**	**CI_{50}: 0.73 ± 0.01**<br>**CI_{75}: 0.70 ± 0.01**<br>**CI_{90}: 0.65 ± 0.04**<br>DRI_{50}CDDP: 2.88 ± 0.17<br>DRI_{50} GANE: 3.51 ± 0.06	**CI_{50}: 0.60 ± 0.04**<br>**CI_{75}: 0.59 ± 0.03**<br>**CI_{90}: 0.68 ± 0.05**<br>DRI_{50}CDDP: 3.13 ± 0.39<br>DRI_{50} TEMS: 3.02 ± 0.68	**CI_{50}: 0.75 ± 0.02**<br>**CI_{75}: 0.66 ± 0.10**<br>CI_{90}: 0.74 ± 0.08<br>DRI_{50} GANE: 2.40 ± 0.014<br>DRI_{50} TEMS: 2.34 ± 0.39	**CI_{50}: 0.59 ± 0.10**<br>**CI_{75}: 0.67 ± 0.07**<br>**CI_{90}: 0.63 ± 0.09**<br>DRI_{50}CDDP: 3.49 ± 0.18<br>DRI_{50} GANE: 5.63 ± 1.34<br>DRI_{50} TEMS: 3.70 ± 0.26
**TOV-112D<br>Pt-res cl.7**	CI_{50}: 0.76 ± 0.13<br>CI_{75}: 0.75 ± 0.06<br>**CI_{90}: 0.75 ± 0.04**<br>DRI_{50}CDDP: 2.51 ± 0.48<br>DRI_{50} GANE: 2.46 ± 0.22	**CI_{50}: 0.61 ± 0.05**<br>**CI_{75}: 0.64 ± 0.06**<br>**CI_{90}: 0.68 ± 0.02**<br>DRI_{50}CDDP: 2.29 ± 0.06<br>DRI_{50} TEMS: 4.10 ± 0.37	CI_{50}: 0.91 ± 0.07<br>**CI_{75}: 0.76 ± 0.04**<br>CI_{90}: 0.85 ± 0.19<br>DRI_{50} GANE: 2.46 ± 0.32<br>DRI_{50} TEMS: 2.29 ± 0.44	**CI_{50}: 0.67 ± 0.04**<br>**CI_{75}: 0.67 ± 0.07**<br>**CI_{90}: 0.69 ± 0.06**<br>DRI_{50}CDDP: 3.86 ± 0.05<br>DRI_{50} GANE: 4.89 ± 1.02<br>DRI_{50} TEMS: 5.36 ± 1.01
**OVCAR 8**	**CI50: 0.64 ± 0.04**<br>**CI75: 0.63 ± 0.04**<br>**CI90: 0.66 ± 0.04**<br>DRI50CDDP: 2.77 ± 0.94<br>DRI50 GANE: 2.54 ± 0.93	**CI50: 0.57 ± 0.02**<br>**CI75: 0.56 ± 0.03**<br>**CI90: 0.57 ± 0.09**<br>DRI50CDDP: 2.15 ± 0.26<br>DRI50 TEMS: 7.57 ± 1.79	**CI50: 0.50 ± 0.02**<br>**CI75: 0.56 ± 0.04**<br>**CI90: 0.70 ± 0.06**<br>DRI50 GANE: 2.58 ± 0.03<br>DRI50 TEMS: 8.49 ± 1.48	**CI50: 0.58 ± 0.02**<br>**CI75: 0.58 ± 0.01**<br>**CI90: 0.66 ± 0.005**<br>DRI50CDDP: 3.36 ± 1.29<br>DRI50 GANE: 3.01 ± 1.37<br>DRI50 TEMS: 26.73 ± 3.13
**OVCAR 8<br>Pt-res cl.2**	**CI50: 0.65 ± 0.04**<br>CI75: 0.80 ± 0.01<br>CI90: 0.87 ± 0.06<br>DRI50CDDP: 3.74 ± 0.53<br>DRI50 GANE: 2.67 ± 0.61	**CI50: 0.74 ± 0.01**<br>**CI75: 0.60 ± 0.16**<br>**CI90: 0.61 ± 0.11**<br>DRI50CDDP: 2.36 ± 0.20<br>DRI50 TEMS: 3.30 ± 0.74	**CI50: 0.75 ± 0.007**<br>CI75: 0.78 ± 0.03<br>CI90: 0.91 ± 0.08<br>DRI50 GANE: 1.81 ± 0.35<br>DRI50 TEMS: 6.65 ± 1.33	**CI50: 0.59 ± 0.08**<br>**CI75: 0.57 ± 0.02**<br>**CI90: 0.58 ± 0.005**<br>DRI50CDDP: 4.82 ± 0.47<br>DRI50 GANE: 2.36 ± 0.63<br>DRI50 TEMS: 11.01 ± 2.42

Cell growth assessment was done by sulforhodamine B colorimetric assay (see Methods). CI values (mean±SD) from at least three separate experiments performed in quadruplicate computed at 50% (CI50), 75% (CI75) and 90% (CI90) of cell kill by CalcuSyn software (Biosoft, Cambridge, UK).
CIs values smaller than 0.8 indicate strong synergism highlighted **in bold**; Cis smaller than 0.9 indicate sinergysm highlighted in bold; additivity (between 0.9 and 1.1) or antagonism (more than 1.1).

DRI values (mean±SD) from at least three separate experiments performed in quadruplicate represent the order of magnitude (fold) of dose reduction obtained for IC50 (DRI50) in combination setting compared with each drug alone.

Supplementary Table 3. Combination index (CI) and dose reduction index (DRI) values for Cisplatin (CDDP), Ganetespib (GANE) and Temsirolimus (TEMS) combinations treatment accordingly to different treatment schedules in **NSCLC models.**

Cell Lines	CDDP+GANE	CDDP+TEMS	GANE+TEMS	CDDP+GANE+TEMS
**A549**	CI_{50}: 0.75 ± 0.07<br>CI_{75}: 0.84 ± 0.02<br>CI_{90}: 0.92 ± 0.06<br>DRI_{50}CDDP: 2.37 ± 0.38<br>DRI_{50} GANE: 2.95 ± 0.41	**CI_{50}: 0.44 ± 0.06**<br>**CI_{75}: 0.38 ± 0.03**<br>**CI_{90}: 0.62 ± 0.04**<br>DRI_{50}CDDP: 7.16 ± 1.48<br>DRI_{50} TEMS: 4.37 ± 1.02	**CI_{50}: 0.60 ± 0.14**<br>**CI_{75}: 0.38 ± 0.05**<br>**CI_{90}: 0.60 ± 0.06**<br>DRI_{50} GANE: 5.50 ± 1.20<br>DRI_{50} TEMS: 2.21 ± 0.79	**CI_{50}: 0.48 ± 0.04**<br>**CI_{75}: 0.41 ± 0.02**<br>CI_{90}: 0.73 ± 0.09<br>DRI_{50}CDDP: 8.83 ± 1.86<br>DRI_{50} GANE: 8.00 ± 1.37<br>DRI_{50} TEMS: 4.88 ± 1.46
**A549 CPr**	**CI_{50}: 0.60 ± 0.07**<br>**CI_{75}: 0.74 ± 0.09**<br>**CI_{90}: 0.67 ± 0.13**<br>DRI_{50}CDDP:6.31 ± 1.17<br>DRI_{50} GANE: 2.14 ± 0.49	CI_{50}: 0.63 ± 0.12<br>CI_{75}: 0.68 ± 0.07<br>CI_{90}: 1.11 ± 0.02<br>DRI_{50}CDDP: 2.86 ± 0.38<br>DRI_{50} TEMS: 4.02 ± 1.54	**CI_{50}: 0.64 ± 0.01**<br>**CI_{75}: 0.64 ± 0.04**<br>CI_{90}: 1.02 ± 0.04<br>DRI_{50} GANE: 2.38 ± 0.28<br>DRI_{50} TEMS: 5.98 ± 2.05	**CI_{50}: 0.50 ± 0.07**<br>**CI_{75}: 0.51 ± 0.06**<br>**CI_{90}: 0.66 ± 0.08**<br>DRI_{50}CDDP: 10.63 ± 1.71<br>DRI_{50} GANE: 3.16 ± 0.66<br>DRI_{50} TEMS: 6.04 ± 0.55

Cell growth assessment was done by sulforhodamine B colorimetric assay (see Methods). CI values (mean±SD) from at least three separate experiments performed in quadruplicate computed at 50% (CI50), 75% (CI75) and 90% (CI90) of cell kill by CalcuSyn software (Biosoft, Cambridge, UK).
CIs values smaller than 0.8 indicate strong synergism highlighted **in bold**; Cis smaller than 0.9 indicate sinergysm highlighted in bold; additivity (between 0.9 and 1.1) or antagonism (more than 1.1).
DRI values (mean±SD) from at least three separate experiments performed in quadruplicate represent the order of magnitude (fold) of dose reduction obtained for IC50 (DRI50) in combination setting compared with each drug alone.

Supplementary Table 4. Sensitivity of HNC cell lines to single agent treatments.

CELL LINES	CDDP<br>IC50 (μM)<br>96h ± SD	GANE<br>IC50 (nM)<br>96h ± SD	TEMS<br>IC50 (nM)<br>96h ± SD
**Cal 27**	1.40 ± 0.20	7.93 ± 1.65	9.75 ± 3.25
**Cal 33**	1.37 ± 0.28	7.53 ± 0.39	4.25 ± 0.72

Abbreviations: CDDP: Cisplatin; GANE: ganetespib; TEMS: Temsirolimus.

IC: Inhibitory Concentration. The IC_{50} values were computed at 96h of treatment (mean±SD) from at least three separate experiments performed in triplicates. Cell growth assessment was done by sulforhodamine B colorimetric assay (see Materials and Methods).

Supplementary Table 5. Combination index (CI) and dose reduction index (DRI) values for Cisplatin (CDDP), Ganetespib (GANE) and Temsirolimus (TEMS) combinations treatment accordingly to different treatment schedule in HNC models.

Cell Lines	CDDP+GANE	CDDP+TEMS	GANE+TEMS	CDDP+GANE+TEMS
**Cal 27**	**CI_{50}: 57 ± 0.07**<br>**CI_{75}: 0.60 ± 0.05**<br>**CI_{90}: 0.63 ± 0.03**<br>DRI_{50}CDDP: 2.62 ± 0.38<br>DRI_{50} GANE: 4.01 ± 0.62	**CI_{50}: 0.53 ± 0.05**<br>**CI_{75}: 0.50 ± 0.03**<br>**CI_{90}: 0.58 ± 0.09**<br>DRI_{50}CDDP: 2.57 ± 0.38<br>DRI_{50} TEMS: 6.99 ± 0.57	**CI_{50}: 0.55 ± 0.11**<br>**CI_{75}: 0.57 ± 0.12**<br>**CI_{90}: 0.71 ± 0.09**<br>DRI_{50} GANE: 4.07 ± 1.00<br>DRI_{50} TEMS: 4.27 ± 0.33	**CI_{50}: 0.63 ± 0.03**<br>**CI_{75}: 0.61 ± 0.04**<br>**CI_{90}: 0.75 ± 0.02**<br>DRI_{50}CDDP: 3.84 ± 0.08<br>DRI_{50} GANE: 5.80 ± 0.62<br>DRI_{50} TEMS: 6.24 ± 1.36
**Cal 33**	CI_{50}: 0.85 ± 0.07<br>CI_{75}: 0.86 ± 0.06<br>CI_{90}: 0.80 ± 0.07<br>DRI_{50}CDDP: 1.58 ± 0.10<br>DRI_{50} GANE: 3.41 ± 0.58	**CI_{50}: 0.41 ± 0.06**<br>**CI_{75}: 0.34 ± 0.02**<br>**CI_{90}: 0.56± 0.03**<br>DRI_{50}CDDP: 3.87 ± 0.84<br>DRI_{50} TEMS: 4.91 ± 0.46	**CI_{50}: 0.40 ± 0.07**<br>**CI_{75}: 0.32 ± 0.01**<br>**CI_{90}: 0.69 ± 0.08**<br>DRI_{50} GANE: 5.39 ± 1.39<br>DRI_{50} TEMS: 4.40 ± 0.69	**CI_{50}: 0.50 ± 0.09**<br>**CI_{75}: 0.48 ± 0.04**<br>**CI_{90}: 0.73 ± 0.02**<br>DRI_{50}CDDP: 5.58 ± 1.63<br>DRI_{50} GANE: 7.36 ± 0.60<br>DRI_{50} TEMS: 5.42 ± 0.80

Cell growth assessment was done by sulforhodamine B colorimetric assay (see Methods). CI values (mean±SD) from at least three separate experiments performed in quadruplicate computed at 50% (CI50), 75% (CI75) and 90% (CI90) of cell kill by CalcuSyn software (Biosoft, Cambridge, UK).
CIs values smaller than 0.8 indicate strong synergism highlighted **in bold**; Cis smaller than 0.9 indicate sinergysm highlighted in bold; additivity (between 0.9 and 1.1) or antagonism (more than 1.1).
DRI values (mean±SD) from at least three separate experiments performed in quadruplicate represent the order of magnitude (fold) of dose reduction obtained for IC50 (DRI50) in combination setting compared with each drug alone.
