# Supplementary material for: Dual inhibition of mTOR and HSP90 enhances cisplatin efficacy and overcomes resistance in ovarian cancer
Source: Cell Death Dis. 2026 Mar 27;17(1):417. doi: 10.1038/s41419-026-08533-3 (PMC13149855; doi:10.1038/s41419-026-08533-3)
Supplement: Supplementary file 4 — Supplementary Materials and Methods [file 41419_2026_8533_MOESM4_ESM.pdf]

Supplementary materials and methods

Cell culture conditions and cisplatin-resistant cell selection

A549, A549 CPr, Cal27 and Cal33 cells were cultured in Dulbecco's modified Eagle's medium (DMEM), whereas TOV-112D ,TOV-112D Pt-res, OVCAR 8 and OVCAR 8 Pt-res were cultured in RPMI-1640 medium. All media were supplemented with 10% FBS (Cambrex, Verviers, Belgium) heat-inactivated, 50 units per mL penicillin (Cambrex), 500 gmL−1 streptomycin (Cambrex), and glutamine 4 mM. The cells were grown in a humidified atmosphere composed of 95% air and 5% CO_2 at 37 °C.

Label free MS-based phosphoproteomics quantitation

TOV-112D Par and TOV-112D Pt-res cl-7 cells were lysed with 0.2% RapiGest SF (Waters, MA, USA) in 50 mM ammonium bicarbonate (AmB) containing PhosSTOP phosphatise inhibitor (Roche, Basel, Switzerland) and incubated in ice for 2 hours. After, samples were denatured at 80˚C for 15 min in a Thermo-mixer R (Eppendorf, Hamburg, Germany) at a speed of 300 rpm and sonicated in a ultrasonic baths (Argo Lab, Italy) at a frequency of 20 kHz for 3 cycle (10 sec each) until the viscosity of the sample was reduced. After centrifugation for 30 min at 14,000 rmp at 4˚C, the amount of proteins was evaluated by Bradford assay. About 100 µg of total proteins, in a final volume of 25 µl, were reduced by 10 mM dithiothreitol (DTT) at 37˚C for 1 hour and then alkylated by 24 mM iodoacetamide (IAA) at 37˚C for 1 hour in the dark (both from Sigma Aldrich, Merck KGaA, Germany). Afterwards, in solution protein digestion was performed diluting the samples at a 1:1 volume ratio with 0.1% RapiGest SF using Sequencing Grade Modified Trypsin at a 1:50 ratio w/w (Promega Corporation, Madison, WI, USA).

Peptide samples were loaded onto a Pepmap precolumn (2 cm × 100 µm, 5 µm, Thermo Fischer Scientific, CA, USA), followed by separation on 25 cm Nano column (0.075µm, Acclaim PepMap100, C18, Thermo Fischer Scientific, CA, USA), at a flow rate of 300 nL/min. Multistep 360-min gradients of ACN were used. The ion trap mass spectrometer equipped with an on-line nanoBoosterCaptiveSpray™ ESI source (Bruker Daltonics, Bremen, Germany) was operated in data-dependent-acquisition mode. To identify phosphorylation sites, two types of peptide fragmentation were carried out in parallel in the mass spectrometer: (i) Collision Induced Dissociation (CID); (ii) Electron Transfer Dissociation (ETD). When CID was used a MS2 was automatically performed on the three most intense MS ions, and MS3 was triggered if one of the top three MS2 peaks corresponded with neutral loss (NL) of 98.0, 49.0, 32.7 m/z. The obtained chromatograms were elaborated using Compass DataAnalysisTM v.4.2 (Bruker Daltonics, Bremen, Germany) and the resulting mass lists were processed using an in-house Mascot search engine (v.2.7.0). Database searching was restricted to the human Swissprot database (accessed April 2021). Trypsin as an enzyme, carbamidomethyl (C) as a fixed modification, oxidation (M), Phospho S-T-Y as variable modifications were set in search parameters. Mass tolerance for all identifications was generally fixed at 2 Da for the precursor ions and 0.8 Da for the product ions. Data were filtered using a global FDR <5% and only proteins with at least one unique identical peptide sequence (p-value < 0.05) were considered identified (1).

Phosphoproteomics quantification analysis

Progenesis QI for proteomics v. 4.2 (Non-linear Dynamics, Newcastle, England) was used as label-free quantification platform. Raw data were imported and the ion intensity maps of all runs (3 for TOV-112D Par and 3 TOV-112D Pt-res cl-7 cells) used for the alignment process to compensate for between-run variation in the LC separation technique. For generate the maximal overlay across the data, only alignment scores above 60% were accepted. Peak peaking was performed using the default sensitivity and a peak width of 0.15 min and charge states of +2, +3 +4 were setting. The survey scan

data is used for the quantification of peptide ions without MS/MS data. Data is then normalized to all proteins. Protein identification was achieved using an in-house Mascot search engine. Protein abundance was calculated using the sum of all unique peptide normalized ion abundances for that protein on each run. Fold changes were calculated selecting only non conflicting peptides (unique) in order to provide a more confidently unambiguous read-out of protein abundance, preventing the overlapping of trends derived from different proteins that shared the same peptides. Statistical tools were used to evaluate the quantitative differences between groups. To indicate the statistical significance of them in group expression data, Anova test (p-value ≤ 0.05) and a fold change of 1.5 was applied. For the power analysis and the estimation of sample size, a threshold of 80% was chosen. Moreover, to afford the multiple testing problem, the FDR adjusted p-values, named q-value, is also provided (q-value ≤0.01). The filtered MASCOT search results were imported back into Progenesis and quantification results were exported and used for further analysis.

Immunoblotting

Primary antibodies were purchased as follows: HSP90 alpha 2G5.G3 (#SMC147) and HSP90 (total) 4F3.E8 (#SMC-149) from StressMarq Biosciences (Victoria, BC, Canada); cleaved PARP1 (Asp214; #5625), PARP1 (#9542), DYRK2 total (#11921), pTHR389-p70S6K (#9205) from Cell Signaling Technology (Leiden, Netherlands); pS2448-mTOR (AB109268); mTOR total (AB32028), p70S6K total (#AB32529), HSP40 (AB223607), His tag (AB18184) from Abcam (Cambridge, UK); β-actin C4 (sc-47778), RPS6 total (SC-74459), from Santa Cruz Biotechnology Inc., (Dallas, TX, USA); ɣH2AX (Ser139) clone JBW301 (#05-636) from Millipore (Burlington, MA, USA); pTYR386/268-DYRK2 (PA5-64575), HSP70 (MA1-10889)from Invitrogen (Waltham, MA, USA); pS326-HSF1 (MAB20526); HSF1 total (MAB16617), AKT total (H00000207-M03) from Abnova (Taipei, Taiwan); pS473-AKT (GTX128414), pS235/236RPS6 (GTX130430), HSP90β (GTX101448) Genetex (Irvine, CA, USA); p^{S241}-PDK1 (TA325773) Origene (Rockville, MD, USA); PDK1 total (E-AB-32535) Elabscience (Huoston, TX, USA). Secondary antibodies were purchased as follows: polyclonal swine anti-rabbit immunoglobulins/horseradish peroxidase (HRP)-linked IgG secondary antibody conjugate and polyclonal rabbit anti-goat immunoglobulins/HRP conjugate from Dako Products, Agilent (Santa Clara, CA, USA); rabbit polyclonal antimouse IgG H&L (HRP) conjugate from Abcam (Cambridge, UK).

Colony formation assay

EOC cell lines (500 cells) and NSCLC cell lines (800 cells) were plated in 6-well plates. Cells were untreated or treated with CDDP, ganetespib and temsirolimus alone or in combination withIC_{10}^{96h} doses of parental cells and were allowed to grow for about 10 days until colonies could be clearly seen. Cell culture plates containing colonies were gently washed with PBS and fixed and stained with crystal violet 0.5%, methanol 20% for 30 min. Excess stain was removed by washing repeatedly with distilled water. Crystal violet was eluted with 100% methanol. Absorbance was read at 595 nm. All the procedures were done at room temperature.

Apoptosis assays

Apoptosis and necrosis were identified by flow cytometry analysis. Briefly, 3×10^5 cells were seeded and after 24h, 48h or 72h of treatment were stained with Annexin VFITC and propidium iodide, following the manufacturer's instructions (MACS; MiltenyiBiotec, Bergisch Gladbach, Germany) and analyzed by flow cytometry.

Plasmid and transient transfection

Human *HSP90α* (*HSP90AA1*) cDNA (Flag-tagged) clone (Catalog Number HG11445-CH) and Negative Control Vector (empty vector, Catalog Number CV015) were obtained from Sino Biological (Beijing, China) and used for transient overexpression experiments. TOV-112D cells were transiently transfected with either the empty vector or the Flag-HSP90α plasmid using Lipofectamine 3000 (Thermo Fisher Scientific) according to the manufacturer's protocol. Forty-eight hours after transfection, cells were collected and used for functional analyses, including clonogenic assays, Annexin V-FITC/propidium iodide staining to assess apoptosis and necrosis, and Western blot analysis of γH2AX expression. Overexpression of HSP90α was verified by Western blot using anti-Flag antibody, with β-actin as loading control.

1. Liu X, Chinello C, Musante L, Cazzaniga M, Tataruch D, Calzaferri G, et al. Intraluminal proteome and peptidome of human urinary extracellular vesicles. Proteom Clin Appl. 2015;9(5–6):568–73.
