# Supplementary figures and images for: Dual inhibition of mTOR and HSP90 enhances cisplatin efficacy and overcomes resistance in ovarian cancer
Source: Cell Death Dis. 2026 Mar 27;17(1):417. doi: 10.1038/s41419-026-08533-3 (PMC13149855; doi:10.1038/s41419-026-08533-3)

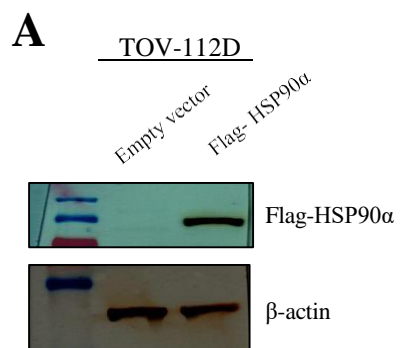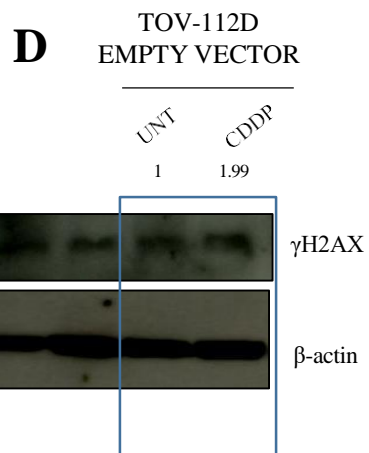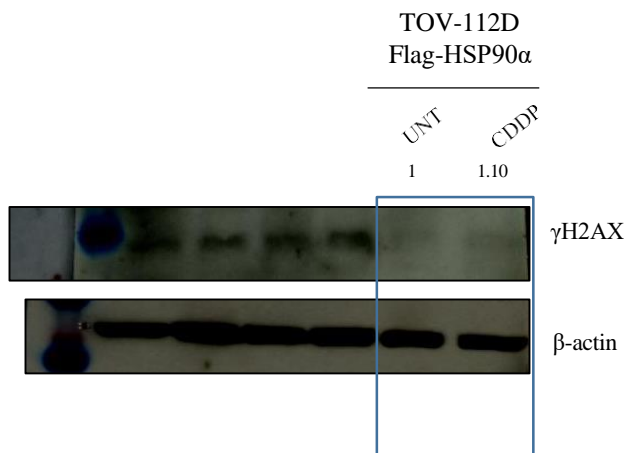

**Figure 3.**

B

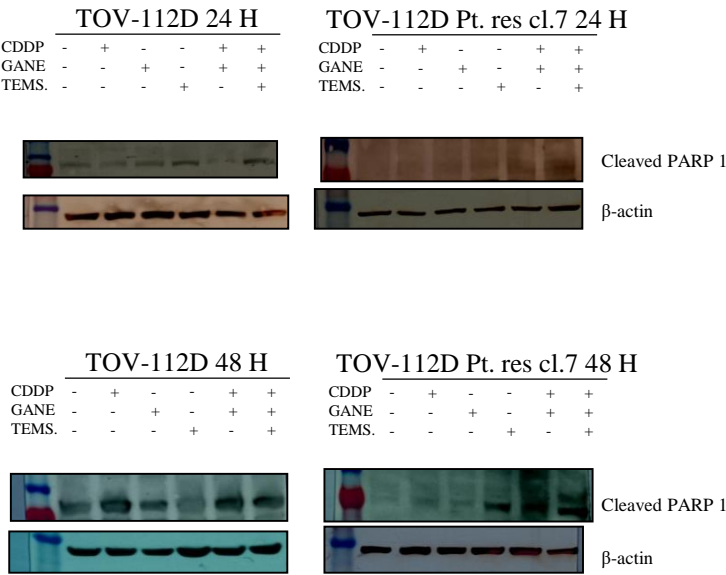

C

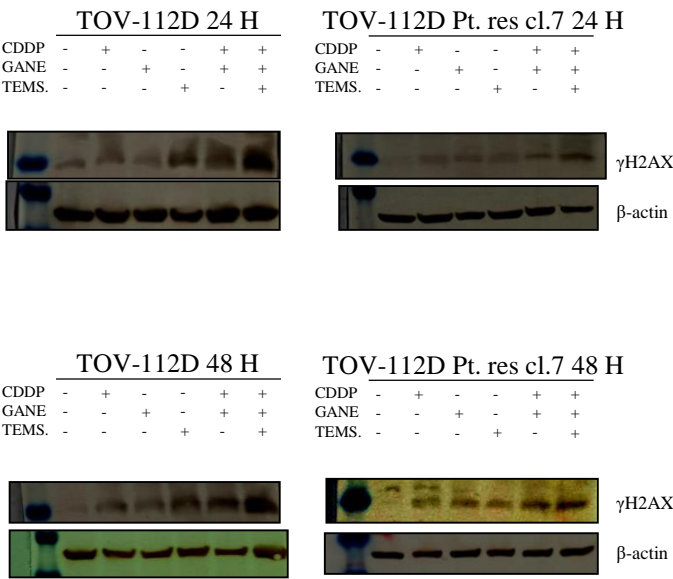

Figure 5.

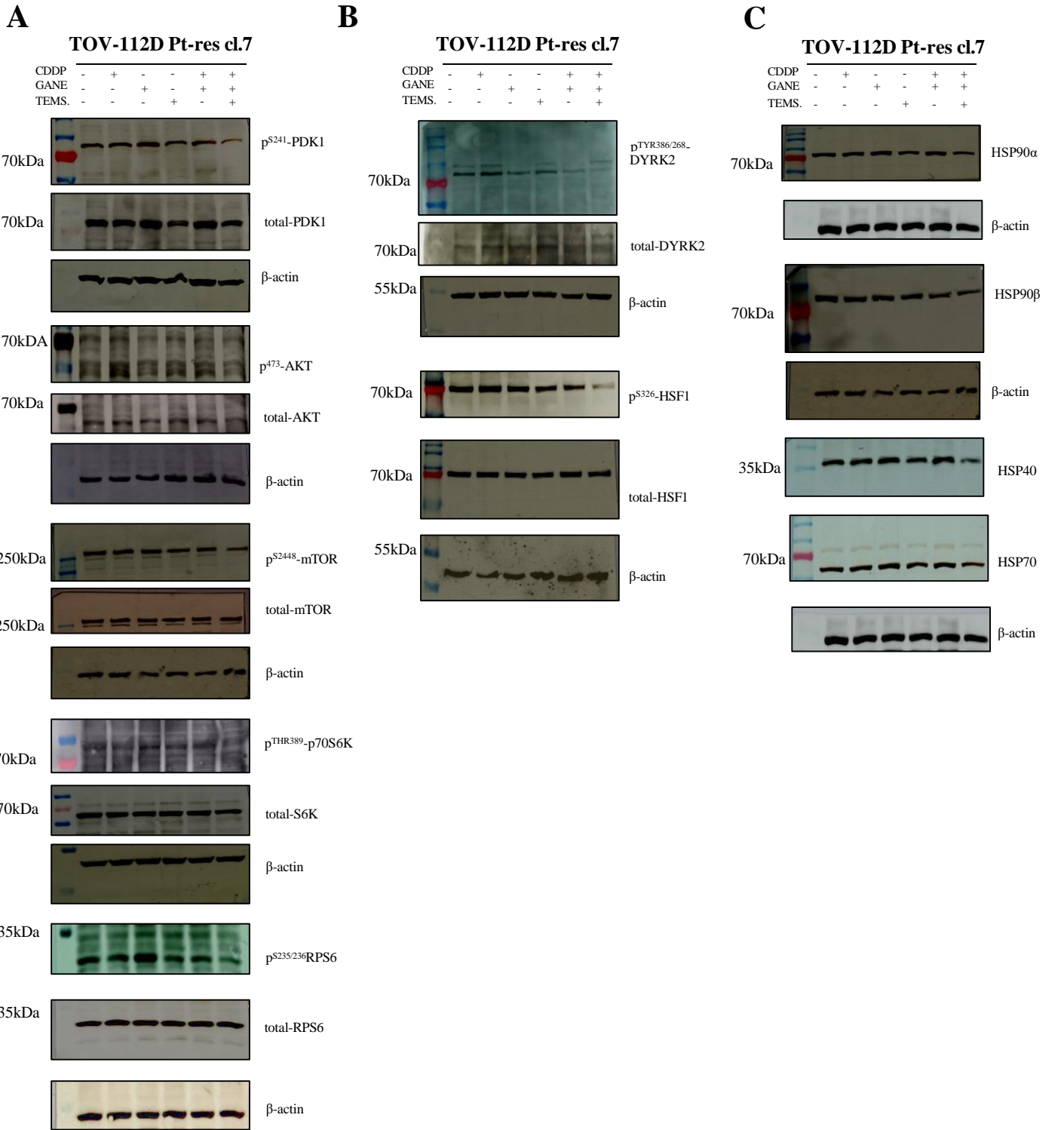

Figure 6.

Supplement: Supplementary file 5 — WB uncropped [file 41419_2026_8533_MOESM5_ESM.pdf]
